# New Multifunctional Agents Based on Conjugates of 4-Amino-2,3-polymethylenequinoline and Butylated Hydroxytoluene for Alzheimer’s Disease Treatment

**DOI:** 10.3390/molecules25245891

**Published:** 2020-12-12

**Authors:** Galina F. Makhaeva, Nadezhda V. Kovaleva, Elena V. Rudakova, Natalia P. Boltneva, Sofya V. Lushchekina, Irina I. Faingold, Darya A. Poletaeva, Yuliya V. Soldatova, Raisa A. Kotelnikova, Igor V. Serkov, Anatoly K. Ustinov, Alexey N. Proshin, Eugene V. Radchenko, Vladimir A. Palyulin, Rudy J. Richardson

**Affiliations:** 1 Institute of Physiologically Active Compounds Russian Academy of Sciences, 142432 Chernogolovka, Russia; gmakh@ipac.ac.ru (G.F.M.); kovalevanv@ipac.ac.ru (N.V.K.); rudakova@ipac.ac.ru (E.V.R.); boltneva@ipac.ac.ru (N.P.B.); sofya.lushchekina@gmail.com (S.V.L.); serkoviv@mail.ru (I.V.S.); ustinov_51@mail.ru (A.K.U.); proshin@ipac.ac.ru (A.N.P.); genie@qsar.chem.msu.ru (E.V.R.); vap@qsar.chem.msu.ru (V.A.P.); 2 Emanuel Institute of Biochemical Physics Russian Academy of Sciences, 119334 Moscow, Russia; 3 Institute of Problems of Chemical Physics of Russian Academy of Sciences, 142432 Chernogolovka, Russia; ifaingold@mail.ru (I.I.F.); dapol@icp.ac.ru (D.A.P.); soldatovayv@gmail.com (Y.V.S.); rkot@icp.ac.ru (R.A.K.); 4 Department of Chemistry, Lomonosov Moscow State University, 119991 Moscow, Russia; 5 Department of Environmental Health Sciences, University of Michigan, Ann Arbor, MI 48109, USA; 6 Department of Neurology, University of Michigan, Ann Arbor, MI 48109, USA; 7 Center of Computational Medicine and Bioinformatics, University of Michigan, Ann Arbor, MI 48109, USA; 8 Michigan Institute for Computational Discovery and Engineering, University of Michigan, Ann Arbor, MI 48109, USA

**Keywords:** 4-amino-2,3-polymethylenequinolines, butylated hydroxytoluene (BHT), acetylcholinesterase (AChE), butyrylcholinesterase (BChE), antioxidants, neuroprotection, ADMET, Alzheimer’s disease (AD)

## Abstract

New hybrids of 4-amino-2,3-polymethylenequinoline with different sizes of the aliphatic ring linked to butylated hydroxytoluene (BHT) by enaminoalkyl (**7**) or aminoalkyl (**8**) spacers were synthesized as potential multifunctional agents for Alzheimer’s disease (AD) treatment. All compounds were potent inhibitors of acetylcholinesterase (AChE) and butyrylcholinesterase (BChE) with selectivity toward BChE. Lead compound **8c**, 2,6-di-*tert*-butyl-4-{[2-(7,8,9,10- tetrahydro-6H-cyclohepta[b]quinolin-11-ylamino)-ethylimino]-methyl}-phenol exhibited an IC_50_(AChE) = 1.90 ± 0.16 µM, IC_50_(BChE) = 0.084 ± 0.008 µM, and 13.6 ± 1.2% propidium displacement at 20 μM. Compounds possessed low activity against carboxylesterase, indicating likely absence of clinically unwanted drug-drug interactions. Kinetics were consistent with mixed-type reversible inhibition of both cholinesterases. Docking indicated binding to catalytic and peripheral AChE sites; peripheral site binding along with propidium displacement suggest the potential of the hybrids to block AChE-induced β-amyloid aggregation, a disease-modifying effect. Compounds demonstrated high antioxidant activity in ABTS and FRAP assays as well as inhibition of luminol chemiluminescence and lipid peroxidation in mouse brain homogenates. Conjugates **8** with amine-containing spacers were better antioxidants than those with enamine spacers **7**. Computational ADMET profiles for all compounds predicted good blood-brain barrier distribution (permeability), good intestinal absorption, and medium cardiac toxicity risk. Overall, based on their favorable pharmacological and ADMET profiles, conjugates **8** appear promising as candidates for AD therapeutics.

## 1. Introduction

Alzheimer’s disease (AD) is a progressive and irreversible neurodegenerative disorder, which is one of the most common forms of dementia in the elderly [1]. It is characterized by progressive impairment of memory and higher cortical functions that lead to the total degradation of mental and intellectual activity, disability, and death [2,3]. There are over 50 million people worldwide with dementia, and the World Alzheimer report for 2019 estimates an increase to more than 152 million cases by 2050 [1].

AD pathogenesis has a multifactorial nature. Although its etiology is unknown, oxidative stress, aberrant protein deposition (β-amyloid and tau protein), cholinergic and glutamatergic mediator systems dysfunctions, and neuronal death appear to contribute to AD pathogenesis [2,4,5,6]. Currently, the anticholinesterase drugs Donepezil (Aricept), Galantamine (Reminyl), and Rivastigmine (Exelon) and the non-competitive NMDA receptor antagonist Memantine are the only drugs used for the symptomatic treatment of AD [7,8]. The anticholinesterase agents compensate the acetylcholine (ACh) deficiency by inhibiting cholinesterases, thereby increasing the duration of ACh action on postsynaptic receptors, enhancing cholinergic transmission, and stimulating cognitive functions. 

Both acetylcholinesterase (AChE, EC 3.1.1.7) and butyrylcholinesterase (BChE, EC 3.1.1.8) inhibitors increase the activation of cholinergic neurons and improve cognitive functions. BChE inhibition is particularly effective with the progression of the disease, when the activity of AChE decreases while the activity of BChE gradually increases [9,10]. Specific BChE inhibition can restore the brain level of acetylcholine in rodents without adverse cholinergic side effects typical of AChE inhibitors [11,12]. It is believed that compounds inhibiting both cholinesterases increase the efficiency of treatment [9,10,13,14]. At the same time, it can be assumed that compounds that are more effective and selective for BChE can enhance cognitive functions, while also minimizing adverse effects in patients with progressive AD.

Aggregation and deposition of β-amyloid (Aβ) peptides in the brain are thought to play key roles in the onset and progression of AD [15,16]. AChE has proaggregant properties toward β-amyloid via involvement of its peripheral anionic site (PAS), which interacts with soluble β-amyloid peptides, thus promoting their aggregation [17,18,19,20,21]. It has been found that PAS ligands, such as propidium, block AChE-induced Aβ aggregation by hampering Aβ binding to the enzyme surface [18,19,22] Moreover, dual-binding molecules that interact with both the catalytic active site (CAS) and the PAS of AChE can inhibit AChE activity and block its amyloidogenic properties. Such compounds could simultaneously improve cognitive function and exert disease-modifying properties [20,23,24,25]. 

Oxidative stress involving an imbalance between the formation of reactive oxygen species (ROS) and their inactivation by various components of the antioxidant system is recognized as a common pathological factor in many neurodegenerative diseases and has been proposed as a general mechanism for age-related degenerative processes. The resultant oxidative damage of membrane lipids and proteins constitutes a vicious cycle of self-propagating injury in the development of neurodegenerative diseases [26,27,28]. Numerous studies have provided compelling evidence linking neuronal oxidative stress to AD, Parkinson’s disease, amyotrophic lateral sclerosis, and multiple sclerosis [29]. It is believed that oxidative stress is associated with such pathological processes as the generation and accumulation of β-amyloid, disturbance of metal ion homeostasis, and mitochondrial dysfunction [30,31]. Accordingly, it would be reasonable to use antioxidants in AD therapy, as well as in development of new multifunctional drugs [29,31,32,33].

Considering the multiplicity of biological pathways involved in AD pathogenesis and progression, the discovery and development of multifunctional, multi-targeted agents with multipronged actions on a number of biological targets involved in AD pathogenesis is both an exceptionally challenging and promising strategy [31,34,35,36,37,38,39]. In contrast to the polypharmacy approach using a combination of drugs, a single drug molecule that could simultaneously attenuate multiple pathogenic pathways would simplify the tasks of optimizing ADMET profiles and reducing the risk of undesirable effects from interactions of individual drug components.

One of the promising approaches in anti-AD drug development is based on multi-target ligands, wherein cholinesterase inhibition is combined with additional biological effects. Attention is devoted to drugs with neuroprotective and disease-modifying properties [40,41,42,43,44,45,46]. Linking two active molecules together through a spacer, resulting in a multifunctional hybrid molecule, is one of the ways to create such multi-target ligands [47]. Tacrine, which was the first FDA-approved drug for AD treatment, is widely employed as an anticholinesterase pharmacophore to create multifunctional cholinesterase inhibitors possessing additional neuroprotective and disease-modifying properties [42,45,48,49,50,51,52,53]. 

**Figure 1:**
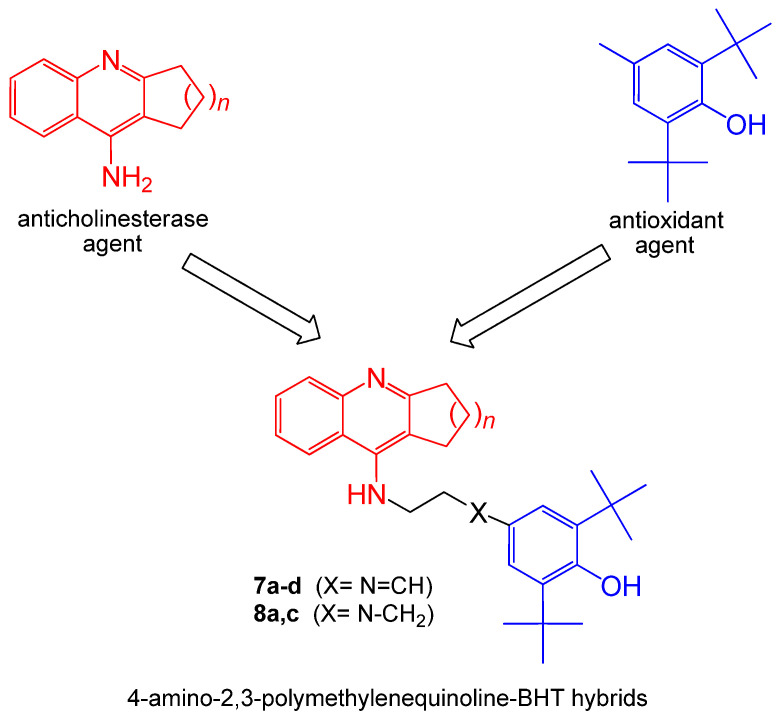
Design strategy of 4-amino-2,3-polymethylenequinolines-BHT hybrids

We applied this approach to create new multifunctional hybrid structures using the 4-amino-2,3-polymethylenequinoline scaffold (which includes tacrine and its cyclic homologues) as the anticholinesterase pharmacophore and the sterically hindered phenolic scaffold of butylated hydroxytoluene (BHT) as the antioxidant pharmacophore (Figure 1).

BHT is a synthetic phenolic antioxidant that has been well-known since 1947 [54]. Later, it was found that this molecule can be generated in nature by some freshwater phytoplankton organisms [55]. Currently, BHT is one of the most commonly used antioxidants in the food, pharmaceutical, and polymer industries [56,57]. BHT and its derivatives have become attractive antioxidant or coantioxidant pharmacophores [58] for the development of new effective antioxidant compounds [57,59,60,61]. In particular, donepezil-BHT hybrids have been recently obtained as potential anti-AD drugs [62]. The optimal hybrid compound had a balanced multifunctional profile including effective inhibition of AChE (IC_50_ = 0.75 μM for hAChE) and monoamine oxidase B (IC_50_ = 7.4 μM), excellent antioxidant activity (0.82 and 1.62 trolox equivalent in ABTS and ORAC methods, respectively), and inhibitory effects on self-induced and AChE-induced Aβ aggregation. Moreover, it was effective and safe in vivo in a scopolamine-induced memory deficit model in mice [62].

Tacrine-antioxidant hybrids (including tacrine-phenolic ones) are being actively developed as powerful cholinesterase inhibitors with additional antioxidant capacity, neuroprotective and disease-modifying properties. These include lipocrine, which combines a derivative of tacrine with lipoic acid to produce a conjugate with extremely potent anti-AChE activity (IC_50_ = 0.253 nM), reduced AChE-induced Aβ aggregation (IC_50_ = 45 μM) and protection of human neuronal cells from ROS formation evoked by oxidative stress [63]. Other examples comprise hybrids of tacrine with ferulic acid [64], melatonin [65], trolox [66], vanillin [67] and many other conjugating moieties [42,68,69].

Here, we describe the synthesis and biological activity evaluation of novel conjugates of 4-amino-2,3-polymethylenequinoline with various sizes of the aliphatic ring, which are connected to BHT by enaminoalkyl or aminoalkyl spacers, as potential multifunctional agents for the treatment of AD. This work included studying the esterase profile of the conjugates, i.e., their inhibitory activity against cholinesterases and a structurally related enzyme, carboxylesterase (CES, EC 3.1.1.1), its analysis based on quantum mechanics (QM)-assisted molecular docking, assessment of propidium displacement from the PAS of AChE from *Electrophorus electricus* (*Ee*AChE) as a measure of their potential ability to block AChE-induced aggregation of β-amyloid, and determination of antioxidant activity. Finally, to assess the potential pharmacokinetic properties of the new compounds, we computationally estimated their profiles for absorption, distribution, metabolism, excretion, and toxicity (ADMET).

## 2. Results and Discussion

### 2.1. Chemistry

**Scheme 1:**
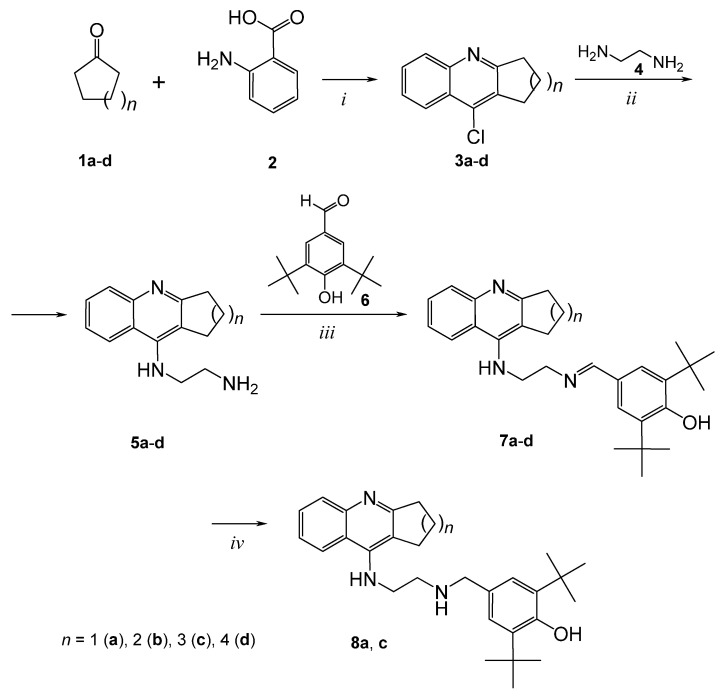
Synthesis of conjugates of 4-amino-2,3-polymethylenequinoline and BHT. *i*: POCl_3_, reflux, 3h; *ii*: ethylenediamine **4**, phenol, 180 °C, 3h; *iii*: aldehyde **6**, toluene, 110 °C, 3h; *iv*: NaBH_4_, MeOH, 25°C, 3h.

We have developed a method for the synthesis of hybrid compounds **7a**–**d** and **8a**, **8c** based on 4-amino-2,3-polymethylenequinoline and containing the antioxidant BHT fragment as the second pharmacophore. Conjugates **7a**–**d** were prepared by the reaction of aminoquinolines **5** with 3,5-di-*tert*-butyl-4-hydroxy-benzaldehyde **6** (Scheme 1). The required aminoquinolines **5** were synthesized by the following method. First, chlorine derivatives **3a-d** were obtained by boiling anthranilic acid **2** with cyclopentanone **1a**, cyclohexanone **1b**, cycloheptanone **1c** or cyclooctanone **1d** in phosphorus oxychloride. Then, the derivatives **3a**–**d** were condensed with ethylenediamine **4** according to the procedure [70] and aminoquinolines **5a**–**d** were obtained. Compounds **8a, 8c** were synthesized by the reduction of the corresponding enamines with NaBH_4_.

Thus, conjugates **7** and **8** with different sizes of the aliphatic ring of 4-amino-2,3-polymethylenequinolines and two types of spacers, enaminoalkyl- and aminoalkyl-containing, have been synthesized. 

### 2.2. Inhibition Studies of AChE, BChE and CES. Structure-Activity Relationships

Assessment of the esterase profile of new potential anti-AD molecules enables one to estimate both the primary pharmacological effects of the tested compounds—their inhibition of AChE and BChE, and their possible unwanted side effects—inhibition of CES that hydrolyzes numerous ester-containing drugs [46,71,72,73]. For the esterase profile evaluation, AChE from human erythrocytes was used along with two enzymes of non-human origin, because of their relatively low cost and the exploratory character of this work. High protein sequence identities between human and equine BChE (90%) and human CES1 and porcine liver CES (77%) (determined using NCBI protein BLAST, http://blast.ncbi.nlm.nih.gov/Blast.cgi?PAGE=Proteins) [71] along with good correlations with results obtained using enzymes from the same species [74] support the applicability of this set of enzymes for determining esterase profiles of new compounds.

The inhibitory activities of the conjugates against the esterases were characterized as IC_50_ values, i.e., the inhibitor concentrations required to decrease a given enzyme activity by 50%. Tacrine, an effective AChE and BChE inhibitor, and bis-4-nitrophenyl phosphate (BNPP), a selective CES inhibitor, were used as reference compounds. The results are shown in Table 1.

Esterase profile studies showed that novel conjugates **7** and **8** effectively inhibit both cholinesterases, although their activity is weaker than that of the basic pharmacophore tacrine (Table 1). Like tacrine, the conjugates inhibit BChE more effectively than AChE. 

Increase of an aliphatic ring size of the 4-amino-2,3-polymethylenequinoline fragment (i.e., the ”tacrine” moiety) from C-5 to C-7 had no effect on anti-AChE activity of conjugates **7**, whereas its increase to C-8 (**7d**) reduced the potency of AChE inhibition ten times. Conjugate **7c** with a cycloheptaquinoline moiety (C-7) exhibited maximum activity toward BChE. Similar effects have been obtained for other hybrid structures based on tacrine cyclic analogs [52,75,76].

Replacing the enamine spacer with an amine one slightly improved the anti-AChE activity and, to a greater extent, affected the increase in the inhibitory activity against BChE. Compound **8c** with a cycloheptaquinoline moiety and an amine spacer was the most potent AChE and BChE inhibitor among the studied conjugates, being only three times less active than tacrine and more selective toward BChE.

As can be seen from Table 1, the novel conjugates possessed rather low activity against CES, the enzyme responsible for the hydrolysis of numerous ester-containing drugs [73]. This is a desirable result, because the inhibition of CES by anticholinesterase compounds used by a patient may lead to unwanted drug-drug interactions [46,72,77].

### 2.3. Kinetic Studies of AChE and BChE Inhibition

**Figure 2:**
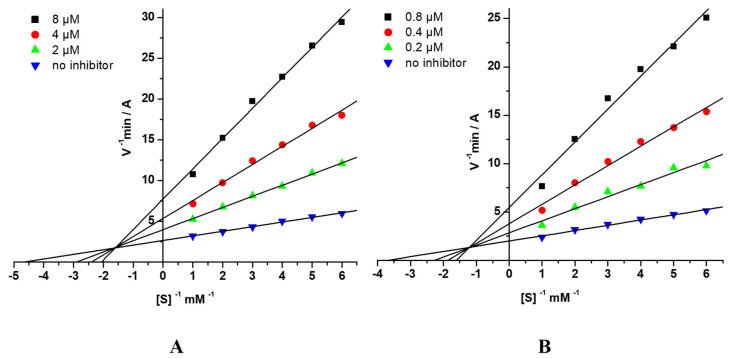
Steady state inhibition of (**A**) AChE and (**B**) BChE by compound **7c**. Lineweaver-Burk double-reciprocal plots of initial velocity and substrate concentrations in the presence of inhibitor (three concentrations) and without inhibitor are presented. [S], mM—concentration of substrates; V—initial velocity rate; A—absorbance.

The mechanism of AChE and BChE inhibition by the conjugates of 4-amino-2,3-polymethylenequinolines and BHT was determined using compound **7c.** The graphical analysis of the kinetic data on AChE (Figure 2A) and BChE (Figure 2B) inhibition by **7c** in the Lineweaver–Burk double-reciprocal plots demonstrated changes in both *K*_m_ and *V*_max_. These results indicate a mixed type of reversible inhibition, with *K*_i_ = 1.58 ± 0.10 μM (competitive component) and *αK*_i_ = 4.30 ± 0.02 μM (noncompetitive component) for AChE. The corresponding constants for BChE were *K*_i_ = 0.164 ± 0.005 μM and *αK*_i_ = 0.437 ± 0.010 μM. 

### 2.4. Molecular Docking Studies

**Figure 3:**
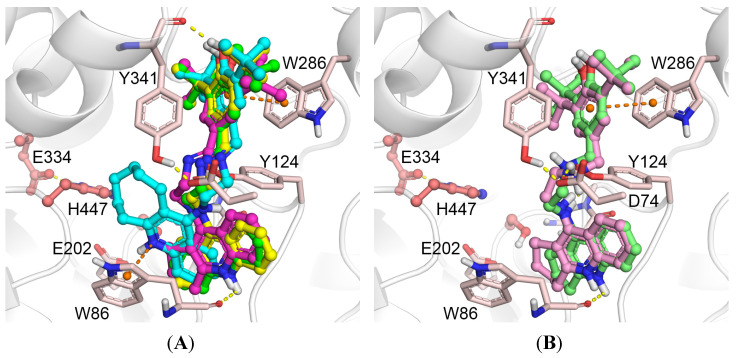

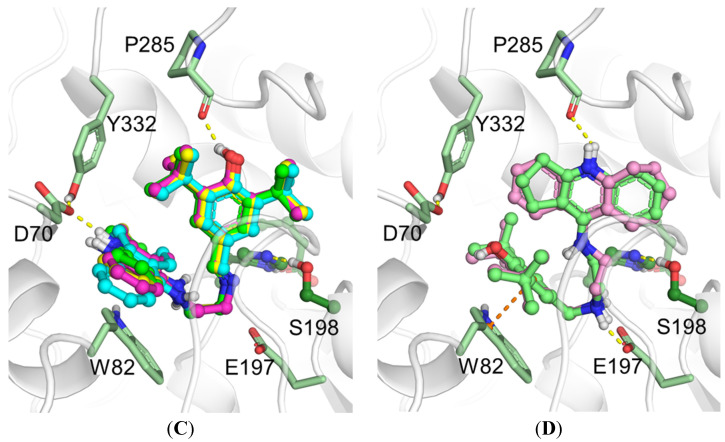
Binding poses obtained by molecular docking with human AChE and compounds **7a**–**d** (**A**), **8a** and **8c** (**B**); with BChE and compounds **7a**–**d** (**C**), **8a** and **8c** (**D**). Carbon atoms of AChE are shown pink, BChE green, **7a** bright green, **7b** yellow, **7c** magenta, **7d** cyan, **8a** pale green, **8c** pale magenta. Yellow dashed lines show hydrogen bonds, orange dashed lines show π-stacking interactions.

Molecular docking of compounds **7a**–**d** to human AChE showed that they bind both to the CAS with the cyclopolymethylenequinoline fragment and to the PAS with the BHT fragment (Figure 3A). In the PAS, binding of the BHT fragment was very similar for all compounds, with a hydrogen bond between the hydroxyl group of the ligand and the backbone oxygen of Tyr341, and π-π stacking interactions with the Trp286 side chain. These interactions of the BHT group with the PAS residues appear to be rather strong, and molecular docking showed that increasing the linker length of compounds **7c** and **8c** to 3- and 4-carbon atoms did not affect the position of the BHT group (see Supplementary Materials Figure S1 A,B). Thus, in case of these hybrids we do not expect a significant increase in anti-AChE activity and propidium displacement with an increase of linker length.

In the CAS, the position of the cyclooctaquinoline fragment (**7d**) was different from that of the other compounds. For compounds **7a**–**c**, hydrogen bonds were observed between the protonated nitrogen atom of the “tacrine” moiety and the backbone oxygen of Trp86 as well as between the external amino group of the “tacrine” moiety and Tyr124. For compound **7d**, the cyclooctaquinoline fragment was displaced due to the large size of the aliphatic ring. Instead, a π-cation interaction between the protonated cyclooctaquinoline group and the Trp86 side chain was observed; however, the cyclooctaquinoline group was too far from Glu202 to form ionic interactions. This explains the decrease in inhibitory activity observed experimentally. The positions of the amine derivatives **8a** and **8c** inside the human AChE gorge did not differ significantly (Figure 3B). The positively charged amino group of the spacer forms a salt bridge with the Asp74 side chain, but this leads to disruption of the hydrogen bond with the backbone oxygen of Tyr341; thus, it does not result in a significant increase in inhibitory activity toward AChE. 

In the case of BChE, no significant differences in binding poses of compounds **7a**–**d** were observed (Figure 3C). All of them shared the same interactions: the hydroxyl group of the BHT fragment formed a hydrogen bond with the backbone oxygen of Pro285, and the charged “tacrine” fragment was involved in ionic interactions with the Asp70 side chain. The binding mode of compounds **8a** and **8c** was different (Figure 3D), with the amino group of the spacer forming an ion pair with the Glu197 side chain. The BHT fragment was parallel to the Trp82 indole ring, suggesting π-π stacking interactions. The “tacrine” fragment formed a hydrogen bond with the backbone oxygen of Pro285. Together, these interactions improved the inhibitory activity of compounds **8a** and **8c** toward BChE compared to compounds **7a**–**d** with an enamine group in the spacer. According to the molecular docking results, an increase of linker length of compounds **7c** and **8c** to 3- and 4-carbon atoms does not appreciably affect the position of the compounds inside the BChE gorge (see Supplementary Materials Figure S1 C,D). Thus, we do not expect a significant increase of anti-BChE activity with an increase of linker length.

### 2.5. Displacement of Propidium Iodide from the PAS of EeAChE

For all conjugates **7** and **8**, the ability to competitively displace propidium from the PAS of *Ee*AChE was evaluated. Propidium is a selective ligand for the PAS of AChE responsible for Aβ binding [17,21,22,78], which showed a significant decrease in AChE-induced Aβ aggregation (82% at 100 μM) [18]. This effect has served as the basis for the fluorescent evaluation of competitive propidium displacement from the PAS of AChE as a method to assess the ability of compounds to bind PAS, suggesting that they would thereby block the AChE-mediated aggregation of β-amyloid [17,19,20]. Donepezil, a mixed-type AChE inhibitor for which the ability to block AChE-PAS-induced Aβ aggregation has been shown [18], was used here as a reference compound. The results are presented in Table 1. It can be seen that all conjugates **7** and **8** at a concentration of 20 μM reduced fluorescence intensity by 14–18% and displaced propidium from the PAS of AChE more effectively than the reference compound donepezil. The conjugates **8a** and **8c** with an amine spacer were somewhat less effective in comparison with the corresponding analogs **7a** and **7c** with an enamine spacer. 

Taken together, the results from propidium displacement, kinetics, and molecular docking indicate that the conjugates **7** and **8** are double-site binding AChE inhibitors, which bind to the PAS of AChE and, therefore, have the potential to block AChE-induced aggregation of β-amyloid.

### 2.6. Antioxidant activity

Primary antioxidant activity of conjugates **7**, **8** was determined spectrophotometrically with two methods, the ABTS radical-scavenging assay and the Fe^3+^ reducing antioxidant power (FRAP) assay. To obtain information about the antioxidant activity of conjugates in biological systems, we estimated their free radical scavenging activity in mice brain homogenate by chemiluminescence (CL) method and studied their ability to suppress spontaneous lipid peroxidation (LP) by the thiobarbituric acid reactive substances (TBARS) assay. The results are presented in Table 2.

#### 2.6.1. ABTS assay

The ABTS assay allows one to measure the radical-scavenging activity of the compounds. The method is based on the determination of absorbance decrease in solution of a stable cation radical ABTS^•+^ (2,2ʹ-azinobis-(3-ethylbenzothiazoline-6-sulfonic acid)) after its interaction with an antioxidant compound [79]. Trolox is used as a reference antioxidant. The results were expressed as TEAC values (Trolox equivalent antioxidant capacity calculated by dividing the slope of ABTS^•+^ concentration decrease vs. the antioxidant concentration by the slope for the Trolox plot) and IC_50_ values. 

The results (Table 2) show that all conjugates **7** exhibit high ABTS^•+^ scavenging activity exceeding the activity of the standard antioxidants Trolox and BHT. Changes in the size of the aliphatic ring in the “tacrine” fragment of the molecule from C-5 to C-8 have practically no effect on the radical-scavenging activity of the compounds whereas replacement of the enamine-containing spacer by the amine one enhanced radical-scavenging activity (compare **7a** and **8a**, **7c** and **8c**). Moreover, compounds **8a**, **8c** proved to be rather fast antioxidants, the maximum binding of the ABTS radical was observed after 5 min.

#### 2.6.2. FRAP assay

The FRAP (ferric reducing antioxidant power) assay measures the ability of antioxidants to reduce the ferric 2,4,6-tripyridyl-*s*-triazine complex [Fe(TPTZ)_2_]^3+^ to the intensely blue ferrous colored complex [Fe(TPTZ)_2_]^2+^ in acidic medium [80,81]. Trolox is used as a reference antioxidant. As can be seen from Table 2, conjugates **7** and **8** are also quite active in the FRAP test. However, the reducing ability of the conjugates is almost twice lower than that of Trolox, and three times lower than that of BHT. Similar to the ABTS test results, the size of the aliphatic “tacrine” ring also has no effect on the Fe^3+^ reducing ability of the conjugates. The reducing ability of the conjugates is enhanced when enamine spacer was replaced with amine one as in ABTS test, and conjugates with alkylamine spacer also were faster antioxidants. 

The results suggest a significant role of the spacer structure, namely, the presence of an enamine or amine fragment in it, for the primary antioxidant activity of 4-amino-2,3-polymethylenequinoline—BHT conjugates. We tried to explain the observed difference using quantum chemical calculation to estimate the HOMO/LUMO gap and isodesmic semireactions to assess the O-H bond strength. However, both approaches showed no significant difference between conjugates having enamine or amine fragment in the spacer. 

#### 2.6.3. Luminol Chemiluminescence Assay

In this method, luminol is used as a chemiluminescence enhancer. The chemiluminescence assay is based on assessing the reduction in the luminol chemiluminescence mediated by its interaction with free radicals whose formation in the mice brain homogenate was initiated by *tert*-butyl hydroperoxide (TBHP) [82]. Luminol allows detecting hydrogen peroxide (H_2_O_2_), hydroxyl radicals (OH), hypochlorite (ClO^–^), peroxynitrite (ONOO^–^), and lipid peroxyl radicals. Inhibition of luminol chemiluminescence in the presence of 20 μM of tested compounds was considered as a measure of their radical-scavenging activity (Table 2).

As can be seen, all conjugates **7, 8** quenched luminol chemiluminescence by 90–95%, exceeding the effects of standard antioxidants BHT (72%) and Trolox (88%). Size of the aliphatic ring in the “tacrine” fragment and replacement of the enamine-containing spacer by the amine one had no major impact on the antioxidant properties of the tested compounds. 

#### 2.6.4. TBARS Assay

Malondialdehyde (MDA) is one of the most prominent secondary products of lipid peroxidation. The reaction of MDA with thiobarbituric acid (TBA) has been widely used as a sensitive method for LP assay in animal tissues [83]. We studied the impact of conjugates **7**, **8** on formation of TBARS by following the reaction of oxidized lipids with TBA in the spontaneous LP conditions in mice brain homogenate. Inhibition of LP was characterized by IC_50_ values (Table 2).

As can be seen from Table 2, conjugates **7** and **8** effectively inhibit the process of spontaneous LP (inhibition of TBARS formation in vitro). The IC_50_ values for the tested compounds were between 6.23 ± 0.23 μM for **8a** and 19.6 ± 0.8 μM for **7a**, they are several times more effective than Trolox (IC_50_ = 91.8 ± 0.3 μM) and close (**7b**, **8a**) or slightly worse than the parent pharmacophore BHT. 

The size of the aliphatic ring in the “tacrine” fragment seems to have only a slight impact on the antioxidant properties of the tested conjugates. As for the effect of the type of spacer, cyclopentaquinoline derivatives **7a** and **8a** demonstrated the most pronounced enhancing in LP suppression when replacing the enamine-containing spacer with the amine one: IC_50_ = 19.6 ± 0.8 μM for **7a** and 6.23 ± 0.33 μM for **8a**. The same trend toward a higher activity of conjugates with an amine spacer persisted for compounds with a cycloheptaquinoline ring: amine **8c** was more active than enamine **7c**.

Therefore, the high activity of conjugates in the ABTS model system and in both tests in a biological system (mouse brain homogenate), along with similar structure-activity trends for the ABTS^•+^ scavenging and LP suppression assays, allow us to assume a significant role of the radical-scavenging activity of the 4-amino-2,3-polymethylenequinoline-BHT hybrids in the realization of their antioxidant activity.

### 2.7. Predicted ADMET Profiles and PAINS Analysis

The results of the computational estimation of a number of ADMET and physicochemical properties for compounds **7a**–**d** and **8a,c** are shown in Table 3. As can be seen, all the compounds had high predicted values for intestinal absorption, enabling their oral administration. Thanks to rather high predicted blood-brain barrier distribution (permeability) for most of the compounds (brain concentration is about 150–400% of the plasma concentration), sufficient CNS activity can be expected. Both parameters of the cardiac toxicity risk (p*K_i_* and pIC_50_) for all analyzed compounds (5.8–6.4 log units) are in the medium part of their possible range (3–9 log units). The predicted lipophilicities and aqueous solubilities of the compounds are somewhat worse than desirable for potential drug compounds according to the commonly accepted drug-likeness rules of thumb. However, given that the compounds are outside of the model applicability domain, the predicted values are not fully reliable. The integral quantitative estimates of drug-likeness (QED) are in the 0.2–0.4 range. The Pan Assay INterference compoundS (PAINS) filter check for the compounds listed in Table 3 did not identified any structural alerts. Thus, the predicted ADMET, physicochemical, and PAINS properties of the compounds are acceptable for potential lead compounds at the early drug development stages. The conjugates with an amine linker seem more promising. However, additional studies and structure optimization are desirable in order to ensure maximal safety and a good pharmacokinetic profile. 

## 3. Materials and Methods

### 3.1. Chemistry

All solvents, chemicals, and reagents were obtained commercially and used without additional purification. ^1^H-NMR (200 MHz) spectra were recorded on a DPX-200 NMR spectrometer (Bruker, Karlsruhe, Germany) using tetramethylsilane as an internal standard. Chemical shifts, δ, are given in parts per million (ppm), and spin multiplicities are given as s (singlet), br s (broad singlet), d (doublet), t (triplet), q (quartet) or m (multiplet). Coupling constants, *J*, are expressed in hertz (Hz). Melting points were recorded on the Stuart SMP10 Melting Point Apparatus (Stuart, Staffordshire, UK) and are uncorrected. Yields refer to isolated pure products and were not maximized. CHN analysis was performed on the ER-20 analyzer (Carlo-Erba, Val-de-Reuil, France). All compounds exhibited analytical and spectroscopic data that strongly agreed with their expected structures.

### 3.2. Synthesis of Compounds

#### General Procedure for the Preparation of Derivatives **7a**–**d** and **8a**, **8c**

A mixture of aminoquinoline **5a**–**d** (1.0 mmol) and 3,5-di-*tert*-butyl-4-hydroxybenzaldehyde **6** (234 mg, 1.0 mmol) in toluene (10 mL) was stirred for 3h at boiling point. Then, the solution was evaporated under vacuum and the residue was washed with ether, yielding the target product **7a**–**d**.

To a solution of enamine **7a**, **7c** (1.0 mmol) in 5 mL of methanol, 57 mg (1.0 mmol) of sodium borohydride was added and the mixture was stirred for 3h at room temperature. Methanol was evaporated, 15 mL of methylene chloride was added and washed with water (2 × 10 mL). The organic layer was dried over anhydrous sodium sulfate. The drying agent was filtered, the filtrate was evaporated, and the residue was recrystallized to give amine **8a**, **8c**. 

*2,6-Di-tert-butyl-4-{[2-(2,3-dihydro-1H-cyclopenta[b]quinolin-9-ylamino)-ethylimino]-methyl}-phenol* (**7a**). Yellow solid; Yield 71%, m.p. 180–183 °C. ^1^H-NMR (CDCl_3_) δ: 1.46 (c, 18H, 6×CH_3_), 2.14 (p, 2H, *J* = 7.3 Hz, CH_2_), 3.05 (t, 2H, *J* = 7.6 Hz, CH_2_), 3.22 (t, 2H, *J* = 7.3 Hz, CH_2_), 3.81 (m, 2H, CH_2_), 3.87 (m, 2H, CH_2_), 5.43 (bs, 1H, NH), 5.56 (bs, 1H, OH), 7.31 (m, 1H, H_ar_), 7.51 (c, 2H, 2×H_ar_), 7.53 (m, 1H, H_ar_), 7.76 (d, 1H, *J* = 9.1 Hz, H_ar_), 7.90 (d, 1H, *J* = 8.5 Hz, H_ar_), 8.10 (c, 1H, =CH). Anal. Calcd. for C_29_H_37_N_3_O: C, 78.52; H, 8.41; N, 9.47. Found: C, 78.41; H, 8.33; N, 9.52.

*2,6-Di-tert-butyl-4-{[2-(1,2,3,4-tetrahydro-acridin-9-ylamino)-ethylimino]-methyl}-phenol* (**7b**). Brown solid; Yield 81%, m.p. 190–193 °C. ^1^H-NMR (CDCl_3_) δ: 1.48 (c, 18H, 6 × CH_3_), 1.86 (m, 4H, 2 × CH_2_), 2.78 (t, 2H, *J* = 5.4 Hz, CH_2_), 3.05 (t, 2H, *J* = 5.6 Hz, CH_2_), 3.99 (m, 4H, 2×CH_2_), 7.31 (m, 1H, H_ar_), 7.52 (m, 1H, H_ar_), 7.58 (c, 2H, 2×H_ar_), 7.91 (d, 1H, *J* = 8.2 Hz, H_ar_), 8.03 (d, 1H, *J* = 8.6 Hz, H_ar_), 8.17 (c, 1H, =CH). Anal. Calcd. for C_30_H_39_N_3_O: C, 78.73; H, 8.59; N, 9.18. Found: C, 78.81; H, 8.51; N, 9.02.

*2,6-Di-tert-butyl-4-{[2-(7,8,9,10-tetrahydro-6H-cyclohepta[b]quinolin-11-ylamino)-ethylimino]-methyl}-phenol* (**7c**). Brown solid; Yield 75%, m.p. 213–215 °C. ^1^H-NMR (CDCl_3_) δ: 1.54 (c, 18H, 6 × CH_3_), 1.70–2.03 (m, 6H, 3 × CH_2_), 3.04 (m, 2H, CH_2_), 3.22 (m, 2H, CH_2_), 3.61 (m, 2H, CH_2_), 3.80 (m, 2H, CH_2_), 4.71 (bs, 1H, NH), 5.58 (s, 1H, OH), 7.23 (m, 1H, H_ar_), 7.60 (m, 1H, H_ar_), 7.67 (c, 2H, 2 × H_ar_), 7.98 (d, 1H, *J* = 8.4 Hz, H_ar_), 8.04 (d, 1H, *J* = 8.2 Hz, H_ar_), 8.28 (c, 1H, =CH). Anal. Calcd. for C_31_H_41_N_3_O: C, 78.94; H, 8.76; N, 8.91. Found: C, 79.04; H, 8.83; N, 8.82.

*2,6-Di-tert-butyl-4-{[2-(6,7,8,9,10,11-hexahydro-cycloocta[b]quinolin-12-ylamino)-ethylimino]-methyl}-phenol* (**7d**). Brown solid; Yield 78%, m.p. 210–213 °C. ^1^H-NMR (CDCl_3_) δ: 1.34 (m, 2H, CH_2_), 1.48 (c, 18H, 6 × CH_3_), 1.62-1.93 (m, 6H, 3 × CH_2_), 3.02 (t, 2H, *J* = 6.2 Hz, CH_2_), 3.12 (t, 2H, *J* = 5.7 Hz, CH_2_), 3.70 (m, 2H, CH_2_), 3.77 (m, 2H, CH_2_), 4.72 (bs, 1H, NH), 5.53 (s, 1H, OH), 7.37 (t, 1H, *J* = 7.4 Hz, H_ar_), 7.56 (t, 1H, *J* = 7.6 Hz, H_ar_), 7.62 (c, 2H, 2 × H_ar_), 7.95 (d, 1H, *J* = 8.2 Hz, H_ar_), 8.08 (d, 1H, *J* = 8.2 Hz, H_ar_), 8.28 (c, 1H, =CH). Anal. Calcd. for C_32_H_43_N_3_O: C, 79.13; H, 8.92; N, 8.65. Found: C, 79.25; H, 8.81; N, 8.72.

*2,6-Di-tert-butyl-4-{[2-(2,3-dihydro-1H-cyclopenta[b]quinolin-9-ylamino)-ethylamino]-methyl}-phenol* (**8a**). Yellow solid; Yield 67%, m.p. 112–114 °C. ^1^H-NMR (CDCl_3_) δ: 1.44 (c, 18H, 6 × CH_3_), 2.10 (m, 2H, CH_2_), 2.78 (q, 2H, *J* = 7.4 Hz, CH_2_), 3.03 (m, 4H, 2×CH_2_), 3.21 (m, 2H, CH_2_), 3.68 (m, 2H, CH_2_), 5.17 (bs, 1H, NH), 5.63 (bs, 1H, OH), 5.78 (bs, 1H, NH), 7.13 (c, 2H, 2 × H_ar_), 7.34 (t, 1H, *J* = 7.4 Hz, H_ar_), 7.54 (t, 1H, *J* = 7.4 Hz, H_ar_), 7.81 (d, 1H, *J* = 8.5 Hz, H_ar_), 7.90 (d, 1H, *J* = 8.0 Hz, H_ar_). Anal. Calcd. for C_29_H_39_N_3_O: C, 78.16; H, 8.82; N, 9.43. Found: C, 78.23; H, 8.31; N, 9.51.

*2,6-Di-tert-butyl-4-{[2-(7,8,9,10-tetrahydro-6H-cyclohepta[b]quinolin-11-ylamino)-ethylamino]-methyl}-phenol* (**8c**). Yellow solid; Yield 71%, m.p. 70–72 °C. ^1^H-NMR (CDCl_3_) δ: 1.46 (c, 18H, 6 × CH_3_), 1.69–2.01 (m, 6H, 3 × CH_2_), 2,84-3.05 (m, 4H, 2 × CH_2_), 3.18 (m, 2H, CH_2_), 3.38 (m, 2H, CH_2_), 3.75 (s, 2H, CH_2_), 4.60 (br.s, 1H, NH), 5.19 (br. s, 1H, OH), 7.18 (c, 2H, 2 × H_ar_), 7.40 (m, 1H, H_ar_), 7.57 (m, 1H, H_ar_), 7.95 (d, 1H, *J* = 8.4 Hz, H_ar_), 8.03 (d, 1H, *J* = 8.2 Hz, H_ar_). Anal. Calcd. for C_31_H_43_N_3_O: C, 78.60; H, 9.15; N, 8.87. Found: C, 79.54; H, 9.23; N, 8.81.

### 3.3. Biological Assays

#### 3.3.1. Enzymatic Assays

- In vitro AChE, BChE, and CES Inhibition

All experiments were carried out in accordance with the standard protocols approved by IPAC RAS. Human erythrocyte AChE, equine serum BChE, porcine liver CES, acetylthiocholine iodide (ATCh), butyrylthiocholine iodide (BTCh), 5,5’-dithio-bis-(2-nitrobenzoic acid) (DTNB), 4-nitrophenol acetate (4-NPA), tacrine, and BNPP were purchased from Sigma-Aldrich (Saint Louis, MO, USA). The activity of AChE and BChE was determined by the method of Ellman (λ = 412 nm) as described in detail in [52]. The activity of CES was determined spectrophotometrically (λ = 405 nm) by the release of 4-nitrophenol using 4-NPA as a substrate as described in [52]. Measurements were performed with a FLUOStar Optima microplate reader (BMG Labtech, Ortenberg, Germany). Compounds were dissolved in DMSO, the incubation mixture contained 2% solvent. The primary evaluation of the inhibitory activity of the compounds was performed by determining the degree of the enzyme inhibition at a compound concentration of 20 µM. For the most active compounds, the values of IC_50_ (µM) for AChE, BChE, and CES inhibition were determined.

- Kinetic Study of AChE and BChE Inhibition. Determination of Steady-State Inhibition Constants

Mechanisms of human erythrocyte AChE and equine serum BChE inhibition were assessed via a thorough analysis of enzyme kinetics. Residual activity was measured following 5 min incubation at 25°C (for temperature equilibration) with three increasing concentrations of inhibitor and six decreasing substrate concentrations. Substrate was added immediately after the 5 min incubation with inhibitor and the rates of absorption were monitored at 412 nm using a FLUOStar OPTIMA microplate reader. Inhibition constants *K*_i_ (competitive component) and α*K*_i_ (noncompetitive component) were determined by linear regression of 1/V versus 1/[S] double-reciprocal (Lineweaver-Burk) plots. 

#### 3.3.2. Propidium Displacement Studies 

Propidium iodide and donepezil were purchased from Sigma-Aldrich. The ability of the test compounds to competitively displace propidium, a selective ligand of the PAS of AChE, was evaluated by the fluorescence method [78,84]. Electric eel AChE (*Ee*AChE, type VI-S, lyophilized powder, Sigma-Aldrich, Saint Louis, MO, USA) was used owing to its high degree of purification, high activity, and lower cost compared to human AChE. In addition, we performed a 3D alignment of the crystal structures of *Ee*AChE (PDB: 1C2O) and human AChE (PDB: 4EY7) using the MUSTANG procedure [85] in YASARA-Structure 18.4.24 for Windows [86], which showed that the two structures were essentially congruent with an RMSD of 0.623 Å over 527 aligned residues and 88.6% sequence identity. The fluorescence intensity of propidium iodide bound with AChE increased several times, whereas decreased fluorescence intensity of the bound propidium in the presence of the test compounds showed their ability to bind to the PAS of AChE to displace propidium [17,19].

To determine the degree of displacement (% displacement) of propidium from the PAS of *Ee*AChE, the enzyme (final concentration, 7 μM) was incubated with the test compound at a concentration of 20 μM in 1 mM Tris-HCl buffer pH 8.0, 25 °C, for 15 min. Then, propidium iodide solution (final concentration, 8 μM) was added, the samples were incubated for 15 min and the fluorescence spectrum (530 nm (excitation) and 600 nm (emission)) was taken. Donepezil and tacrine were used as reference compounds. The blank contained propidium iodide of the same concentration in 1 mM Tris-HCl buffer pH 8.0. The measurements were carried out in triplicate on a FLUOStar Optima microplate reader, and the results were calculated by formula (1):

% Displacement = 100 − (IF_AChE+ Propidium + inhibitor_ / IF_AChE + Propidium_) × 100 (1)

where IF_AChE + Propidium_ is the fluorescence intensity of the propidium associated with AChE in the absence of the test compound (taken as 100%), and IF_AChE + Propidium + inhibitor_ is the fluorescence intensity of the propidium associated with AChE in the presence of the test compound.

#### 3.3.3. Antioxidant Activity

- ABTS radical cation scavenging activity assay

Radical scavenging activity of the compounds was assessed using the ABTS radical decolorization assay [79] with some modifications [53]. ABTS was purchased from Tokyo Chemical Industry Co., Ltd. (Tokyo, Japan), potassium persulfate (dipotassium peroxodisulfate), Trolox (6-hydroxy-2,5,7,8-tetramethychroman-2-carboxylic acid), and HPLC-grade ethanol were obtained from Sigma-Aldrich. Aqueous solutions were prepared using deionized water.

The solution of cation radical ABTS^•+^ was produced by incubation of ABTS with potassium persulfate in deionized water for 12–16 h at room temperature in the dark. Radical scavenging capacity of the compounds was analyzed by mixing 10 μL of compound with 240 μL of ABTS^•+^ working solution in ethanol (100 μM final concentration). The reaction was monitored for an hour with an interval of 5–10 min. Data are given for 1 h of incubation of compounds with ABTS^•+^. The reduction in absorbance was measured spectrophotometrically at 734 nm using a xMark microplate UV/VIS microplate spectrophotometer (Bio-Rad, Hercules, CA, USA).

Ethanol blanks were run in each assay. Values were obtained from three replicates of each sample and three independent experiments. Standard antioxidants Trolox and BHT were used as reference compounds. 

Antioxidant capacity as Trolox equivalent (TEAC) values was determined as the ratio between the slopes obtained from the linear correlation of the ABTS radical absorbance with the concentrations of tested compounds and Trolox. For the compounds, we also determined the IC_50_ values (compound concentration required for 50% reduction of the ABTS radical). The compounds were tested in the concentration range of 0.1–100 µM.

- FRAP

The FRAP (ferric reducing antioxidant power) assay proposed by Benzie and Strain [80,81] was modified to be performed in 96-well microplates. The FRAP reagent contained 2.5 mL of 10 mM TPTZ (2,4,6-tris(2-pyridyl)-s-triazine (Sigma-Aldrich) solution in 40 mM HCl, 2.5 mL of 20 mM FeCl_3_ (Sigma-Aldrich) in distilled water and 25 mL of 0.3 M acetate buffer (pH 3.6). In 96-well microplates, 10 µL aliquots of the substance under investigation (compounds **7, 8** or reference substance) dissolved in DMSO (0.5 mM) were placed in quadruplicate. The FRAP reagent (240 µL) freshly prepared and warmed to 37°C was added to three of these samples, while the same volume of acetate buffer was added to the fourth one (blank). Consequently, the concentration of the substance in a plate was 20 µM. The absorbance at 600 nm was monitored spectrophotometrically with a FLUOStar OPTIMA microplate reader at 37°C for an hour with an interval of 5–10 min. Data in Table 2 are given for 1 h of incubation of compounds with FRAP reagent. The absorbances of the blanks and those of the mixture of 240 µL FRAP reagent plus 10 µL DMSO were subtracted from the absorbances of the samples at each time interval to calculate the absorbance change. Standard antioxidants Trolox and BHT dissolved in DMSO and analyzed as described above were used as reference compounds. A calibration curve with linear formula y = 0.03x (*R*^2^ = 0.998, *p* < 0.0001) was obtained for Trolox solution in DMSO in the range 2.5–100 µM and the results were expressed as FRAP units (the values calculated as the ratio of the concentrations of Trolox and the test compound resulting in the same effect.)

- Tissue preparation

The work was carried out in accordance with the EU Directive 2010/63/EU. Hybrid BDF1 mice (about 6 months old) were sacrificed by decapitation. Each brain was rapidly excised, frozen in liquid nitrogen, and stored at −80°C until use. The brains were thawed and homogenized using a Wisd WiseTis HG-15D homogenizer for 2 min in a buffer (0.01 M PBS, pH 7.4 or 0.1 M Tris-HCl, pH 7.4). Protein concentrations were determined by the Lowry method [87].

- Luminol Chemiluminescence Assay

Luminol-dependent chemiluminescence produced by mouse brain homogenates was measured using a 1250 Luminometer (LKB Wallac, Turku, Finland) according to the assay by Di Meo et al. [82] with minor modifications. The reaction mixture consisted of mouse brain homogenate (protein concentration 0.1 mg/mL) in 0.1 M Tris-HCl (Sigma-Aldrich), pH 7.4, luminol (Sigma-Aldrich) (0.05 mM), TBHP (Sigma-Aldrich) (0.073 M) and tested compounds at a concentration of 20 μM. This was placed in a light-proof luminometer chamber and the background reading was recorded. All measurements were carried out at 37°C. The gain control was set to give a reading of 10 mV using a built-in standard. The free radical content in mouse brain homogenate was evaluated by the change in the light sum (the area under the kinetic curve of luminescence intensity for the entire luminescence time). All compounds were examined at least in triplicate. The effects of tested compounds were measured as the percentage decrease in the light sum relative to the control (a sample with no compound added).

- TBARS Assay

The procedure was performed in accordance with the method of Ohkawa et al. [83] with minor modifications. Briefly, mouse brain homogenate (protein concentration 1 mg/mL) in PBS (0.1 M, pH 7.4) was incubated for 30 min at 37°C with tested conjugates, and the reaction was terminated by addition of 0.4 mL of 17% (*w*/*v*) trichloroacetic acid (Sigma-Aldrich). Following centrifugation for 20 min at 1300× *g*, 0.5 mL of 0.8% (*w*/*v*) of TBA (Sigma-Aldrich) was added to 1 mL of supernatant, heated for 30 min at 95°C and then cooled to RT. The optical density of the TBARS, which corresponds to the produced MDA, was measured at 532 nm against a blank using an Agilent Cary 60 UV-Vis spectrophotometer (Agilent, Santa Clara, CA, USA). The MDA concentration was calculated using an extinction coefficient of 1.56 × 10^5^ M^−1^cm^−1^. Trolox and BHT were used as reference antioxidants. Compounds were dissolved in DMSO (Sigma-Aldrich) and tested in a concentration range 0.01–100 μM. All compounds were examined at least in triplicate. IC_50_ values represent the concentration that caused 50% inhibition of LP.

### 3.4. Molecular Modeling Studies 

X-ray structures of human AChE co-crystallized with donepezil (PDB: 4EY7, see more details in the Supplementary Materials) [88] and an optimized X-ray structure of human BChE (PDB: 1P0I) [89,90] were used for molecular docking. Ligand structures were optimized using a DFT quantum chemistry method (B3LYP/6-31G*, GAMESS-US [91] software). Partial atomic charges on ligand atoms were assigned from QM data according to the Löwdin scheme [92]. Molecular docking was performed with AutoDock 4.2.6 software [93]. The grid box for docking included the entire active site gorge of AChE (22.5 Å × 22.5 Å × 22.5 Å grid box dimensions) and BChE (15 Å × 20.25 Å × 18 Å grid box dimensions) with a grid spacing of 0.375 Å. The main Lamarckian Genetic Algorithm (LGA) [94] parameters were 256 runs, 25 × 10^6^ evaluations, 27 × 10^4^ generations, and a population size of 3000. Figures were prepared with PyMOL (www.pymol.org).

### 3.5. Prediction of ADMET Profiles and PAINS Analysis

Human intestinal absorption (HIA) [95], blood-brain barrier distribution/permeability (LogBB) [96], and hERG-mediated cardiac toxicity risk (channel affinity p*K*i and inhibitory activity pIC_50_) [97] were estimated using the integrated online service for ADMET properties prediction [98]. This service implements predictive QSAR models based on accurate and representative training sets, fragmental descriptors, and artificial neural networks. Lipophilicity (LogP_ow_) and aqueous solubility (pS) were estimated by the ALogPS 3.0 neural network model implemented in the OCHEM platform [99]. The quantitative estimate of drug-likeness (QED) values [100] were calculated and the PAINS alerts were checked using RDKit version 2020.03.4 software [101].

### 3.6. Statistical Analyses

All tests were performed at least in triplicate in three independent experiments. Results are presented as mean ± SEM calculated using GraphPad Prism version 6.05 for Windows (San Diego CA, USA). Plots, linear regressions, and IC_50_ values were determined using Origin 6.1 for Windows, OriginLab (Northampton, MA, USA). 

## 4. Conclusions

New hybrids of 4-amino-2,3-polymethylenequinoline and BHT with different sizes of the aliphatic ring linked to BHT by enaminoalkyl (**7**) or aminoalkyl (**8**) spacers were synthesized. All compounds exhibited high dual anticholinesterase activity with selectivity toward BChE and mixed-type reversible inhibition of both cholinesterases. They also had rather poor anti-CES activity, suggesting a low tendency to exert potential unwanted drug-drug interactions in clinical use. A lead compound **8c** was identified.

Molecular docking indicated dual binding sites of the conjugates in AChE and the possibility of binding to the AChE PAS that, along with results on propidium displacement, suggest their potential to block AChE-induced Aβ aggregation, thereby exerting a disease-modifying effect.

New conjugates exhibited high ABTS^•+^-scavenging activity exceeding the activity of the standard antioxidants Trolox and BHT and were quite active in the FRAP test. Moreover, replacement of the enamine-containing spacer by the amine-containing one enhanced radical-scavenging activity. The conjugates also were highly active in antioxidant tests in a biological system (mouse brain homogenate), demonstrating high radical scavenging activity exceeding that of BHT and Trolox in the luminol chemiluminescence test and inhibiting spontaneous LP at micromolar IC_50_ values. The results suggest a significant role of the radical-scavenging activity of 4-amino-2,3-polymethylenequinoline-BHT hybrids in the realization of their antioxidant activity.

Computational ADMET profiles predicted good blood-brain barrier distribution (permeability), good intestinal absorption, and medium cardiac toxicity risk for all compounds. The conjugates with an amine spacer appeared more promising as potential AD therapeutics than those with an enamine space in view of their better pharmacological and ADMET profiles. 

Thus, the new conjugates of tacrine homologs and BHT have the potential to treat symptoms of AD as well as to exert disease-modifying and high antioxidant effects, thereby showing promise for further development and optimization as multitarget anti-AD agents.

## Figures and Tables

**Table 1:** Esterase profiles of conjugates **7, 8** and their ability to displace propidium.

Compound		Inhibitory Activity Against AChE, BChE, and CES and Inhibitor Selectivity				Displacement of Propidium from *Ee*AChE PAS (%) ^1^
N	*n*	Human Erythrocyte AChE, IC_50_ (µM)	Equine Serum BChE, IC_50_ (µM)	Porcine Liver CES, (%) ^1^	Selectivity for BChE ^2^	
**7a**	1	4.86 ± 0.01	1.92 ± 0.11	26.1 ± 0.7	2.5	18.1 ± 1.6
**7b**	2	5.98 ± 0.13	1.61 ± 0.04	28.6 ± 1.6	3.7	18.2 ± 1.6
**7c**	3	4.03 ± 0.03	0.419 ± 0.040	30.1 ± 2.5	9.6	16.3 ± 1.3
**7d**	4	45.0 ± 5.7	4.27 ± 0.26	20.4 ± 1.6	10.5	17.1 ± 1.8
**8a**	1	3.50 ± 0.33	0.652 ± 0.005	19.6 ± 1.3	5.4	16.4 ± 1.4
**8c**	3	1.90 ± 0.16	0.084 ± 0.008	26.0 ± 1.9	22.6	13.6 ± 1.2
**Tacrine**		0.601 ± 0.047	0.0295 ± 0.002	n.a.	20.4	4.4 ± 0.6
**BHT**		n.a.	n.a.	n.a.	–	n.d.
**BNPP**		n.a.	n.a.	92.1 ± 1.8 ^3^	n.d.	n.d.
**Donepezil**		0.040 ± 0.004	19.2 ± 3.0	n.a.	0.002	10.1 ± 0.6

^1^ compound concentration 20 µM. ^2^ selectivity for BChE = IC_50_ AChE / IC_50_ BChE. ^3^ BNPP IC_50_ CES = 1.80±0.11 µM. n.d.—not determined. n.a.—not active. Data are expressed as mean ± SEM, *n* = 3.

**Table 2:** Antioxidant properties of conjugates **7** and **8.**

Compound		ABTS^•+^-Scavenging Activity		Ferric Reducing Antioxidant Power	Radical Scavenging Capacity in Luminol Chemiluminescence Assay	Inhibition of Spontaneous Lipid Peroxidation in Mice Brain Homogenate, TBARS Assay
N	*n*	**TEAC** **^1^**	**IC_50_, µM**	**FRAP Units** **^2^**	**Luminescence Inhibition, % ^3^**	**IC_50_** **,** **μM**
**7a**	1	0.92 ± 0.03	22.3 ± 1.5	0.51 ± 0.03	94.0 ± 0.2	19.6 ± 0.8
**7b**	2	1.13 ± 0.05	17.1 ± 1.4	0.58 ± 0.03	95.0 ± 0.2	7.21 ± 0.62
**7c**	3	1.11 ± 0.04	17.8 ± 1.6	0.52 ± 0.04	95.0 ± 0.4	10.4 ± 0.7
**7d**	4	1.12 ± 0.05	17.8 ± 1.7	0.57 ± 0.02	95.0 ± 0.2	14.8 ± 1.3
**8a**	1	1.39 ± 0.05	12.7 ± 1.1	0.77 ± 0.02	95.0 ± 0.1	6.23 ± 0.33
**8c**	3	1.35 ± 0.04	14.1 ± 0.8	0.80 ± 0.06	91.0 ± 0.6	9.38 ± 0.12
**BHT**		0.98 ± 0.03	22.4 ± 1.4	1.76 ± 0.07	72.0 ± 2.6	6.89 ± 0.26
**Trolox**		1.0	20.1 ± 1.2	1.0	88.0 ± 1.0	91.8 ± 0.3

^1^ TEAC (Trolox Equivalent Antioxidant Capacity) was determined from the ratio of the slopes of the concentration-response curves test compound/Trolox. ^2^ FRAP units—Ferric Reducing Antioxidant Power units, the values calculated as the ratio of the concentrations of Trolox and the test compound resulting in the same effect. ^3^ Compound concentration 20 µM. Data are expressed as mean ± SEM, *n* = 3.

**Table 3:** Predicted ADMET and physicochemical profiles of conjugates **7** and **8**.

Compound		LogBB	HIA, %	hERG, *pK_i_*	hERG, *pIC_50_*	LogP_ow_	pS	QED
N	*n*							
**7a**	1	0.19	100	6.22	6.17	5.82	6.82	0.35
**7b**	2	0.26	100	6.27	6.06	6.13	7.19	0.32
**7c**	3	0.33	100	6.27	6.17	6.40	7.43	0.23
**7d**	4	0.38	100	6.37	6.32	6.71	7.68	0.29
**8a**	1	0.48	100	5.87	5.83	5.32	5.67	0.39
**8c**	3	0.61	100	5.93	5.82	5.93	6.28	0.27
**Tacrine**		–0.04	93	4.98	4.98	2.95	1.52	0.71
**BHT**		0.59	100	5.75	5.25	5.31	4.9	0.69

LogBB—blood-brain barrier distribution, HIA—human intestinal absorption [%], hERG *pKi—*hERG potassium channel affinity [−log(M)], hERG pIC_50_—hERG potassium channel inhibitory activity [−log(M)], LogP_ow_—octanol–water partition coefficient, pS—aqueous solubility [–log(M)], QED—quantitative estimate of drug-likeness.

## Supplementary Materials

The following are available online: Supplementary (1) molecular docking studies including Figures S1 and S2 and (2) NMR spectra for **7a**–**d, 8a**, **8c**: Figure S3. NMR Spectra for **7a**; Figure S4. NMR Spectra for **7b**; Figure S5. NMR Spectra for **7c**; Figure S6. NMR Spectra for **7d**; Figure S7. NMR Spectra for **8a**; Figure S8. NMR Spectra for **8c**.
