# Supplementary material for: New Multifunctional Agents Based on Conjugates of 4-Amino-2,3-polymethylenequinoline and Butylated Hydroxytoluene for Alzheimer’s Disease Treatment
Source: Molecules. 2020 Dec 12;25(24):5891. doi: 10.3390/molecules25245891 (PMC7763995; doi:10.3390/molecules25245891)
Supplement: Supplementary file 1 [file molecules-25-05891-s001.zip › Supplementary/1_supplemental_Docking.docx]

**Supplementary molecular docking studies**

| 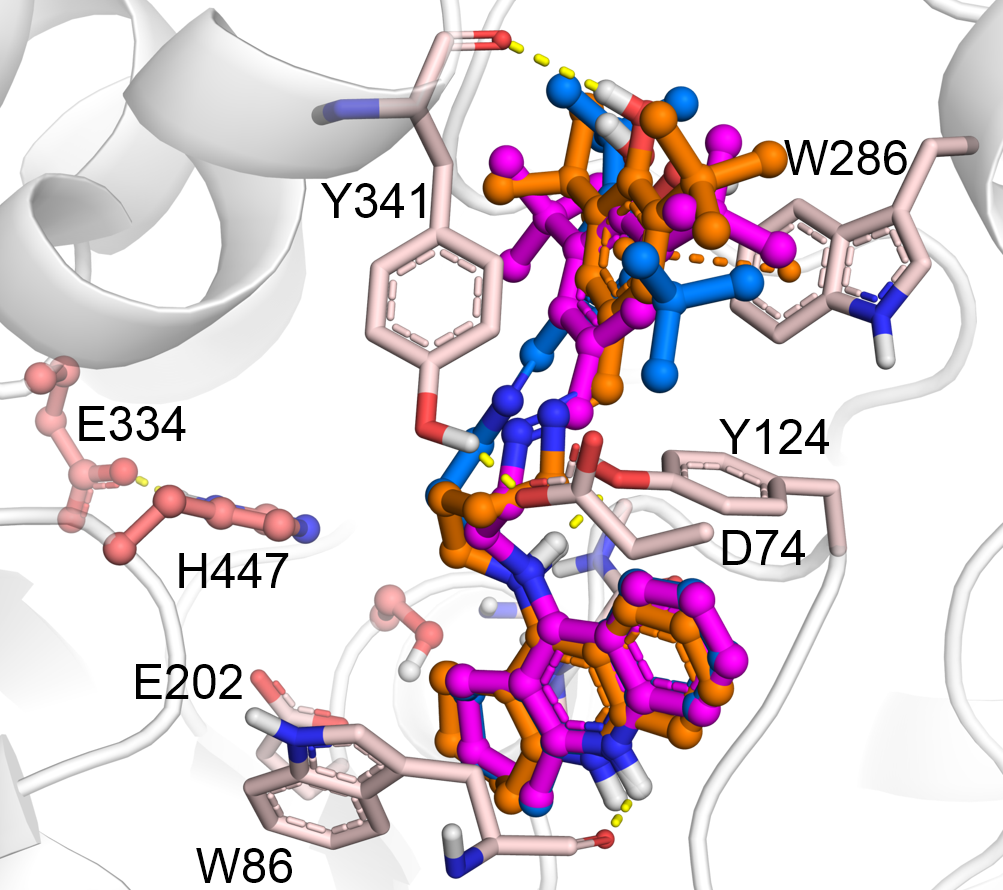 | 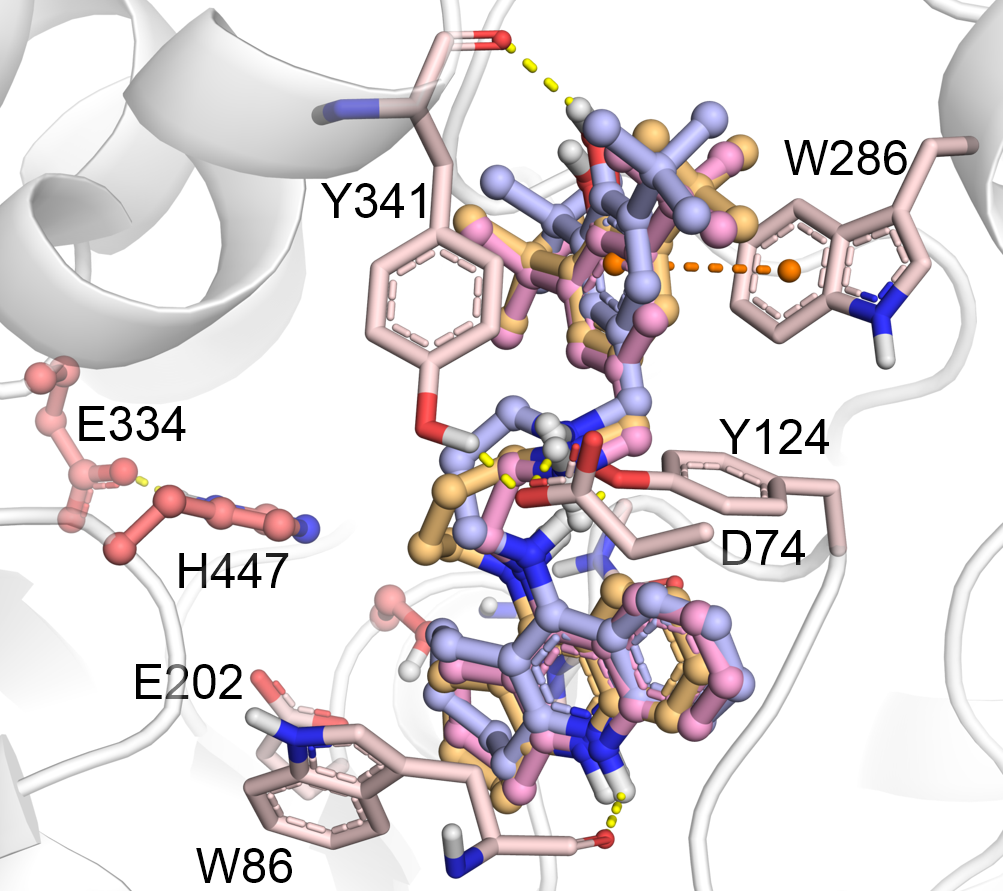 |
| --- | --- |
| **(A)** | **(B)** |
| 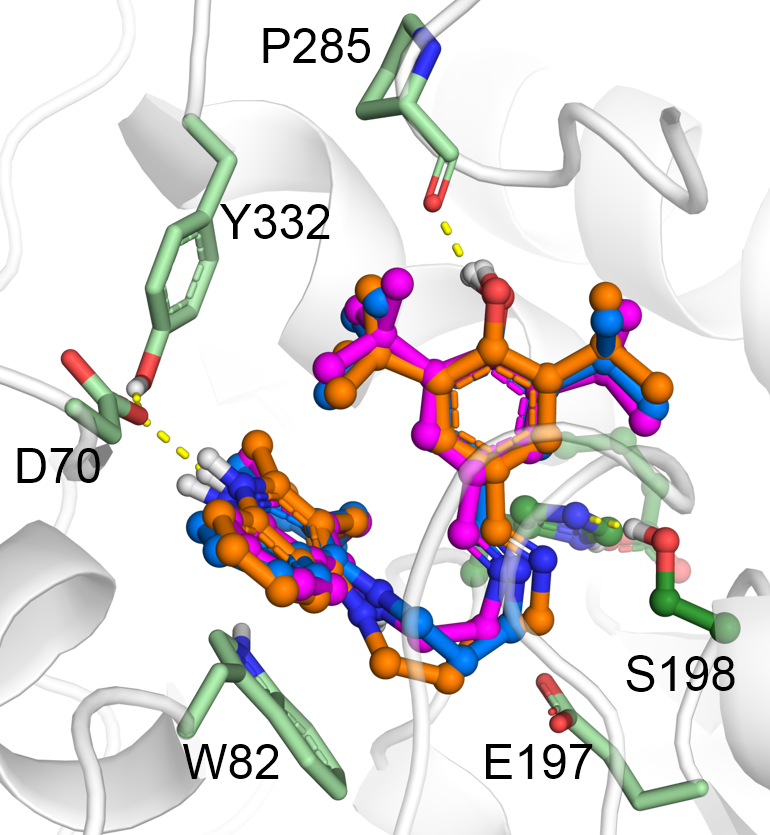 | 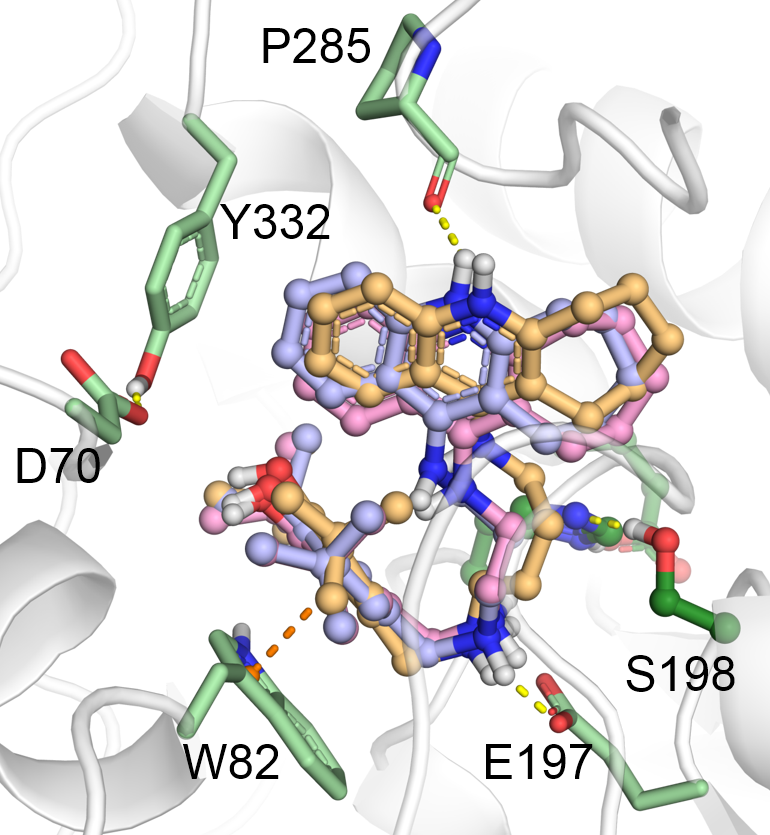 |
| **(C)** | **(D)** |

**Figure S1.** Binding poses obtained by molecular docking of analogs of compounds **7c** (A,C) and **8c** (B,D) containing 3- and 4-carbon linkers (colored blue and orange, respectively) to AChE (A,B) and BChE (C,D).

Our earlier methodological studies showed that the X-ray crystal structure of human AChE co-crystallized with donepezil (PDB ID 4EY7) was better suited than the apo-AChE structure or AChE co-crystallized with other ligands (PDB ID 4EY4-4EY8) for docking of bifunctional compounds containing a linker [1,2]. In the first place, better results of docking of bulky ligands were achieved due to rotation of the Tyr337 side chain induced by donepezil [3,4]. This allowed better accommodation of the linker part of bifunctional inhibitors. Based on those findings we have routinely used this X-ray structure for docking of hybrids. However, it is of some interest to compare docking results with the X-ray crystal structure of AChE co-crystallized with tacrine derivatives.

Among complexes of AChEs with tacrine derivatives deposited in the PDB, there is none of human AChE^1^. The majority are complexes of *Torpedo californica* (*Tetronarce californica*) AChE, which are not ideal because human AChE was used for our experimental kinetic measurements. Indeed, **three orders** of magnitude difference in inhibition constants for hAChE and TcAChE inhibition by rivastigmine [7] is a strong warning against the use of TcAChE X-ray structures to model inhibition of human AChE. With respect to molecular docking, we found that the most important variable was Phe330/Tyr337 replacement of homologous amino acids for Phe330/Tyr337, which played a key role in the binding of ligands in the gorge. Other differences between human and fish AChE sequences mostly affect protein dynamics [8].

However, there are some structures of mouse AChE (mAChE) co-crystallized with tacrine derivatives, which might be used due to the high similarity between human and mouse AChEs. Among those structures of mAChE+tacrine derivatives, some are Y337A mutants [9], and our study emphasized the crucial role of Tyr337 in the binding of such compounds. Therefore, only a few structures are left, and they are complexes of conjugates of tacrine with a propidium-like fragment. The latter induces conformational changes in the PAS. However, there are two structures less affected by binding of the propidium-like fragment, PDB ID 5EHQ and 5EIA [9] we performed docking of compounds **7c** and **8c** with them. Docking results with X-ray structures 5EHQ and 5EIA were identical and very close to those reported in the main text with human AChE (Figure S1).

| 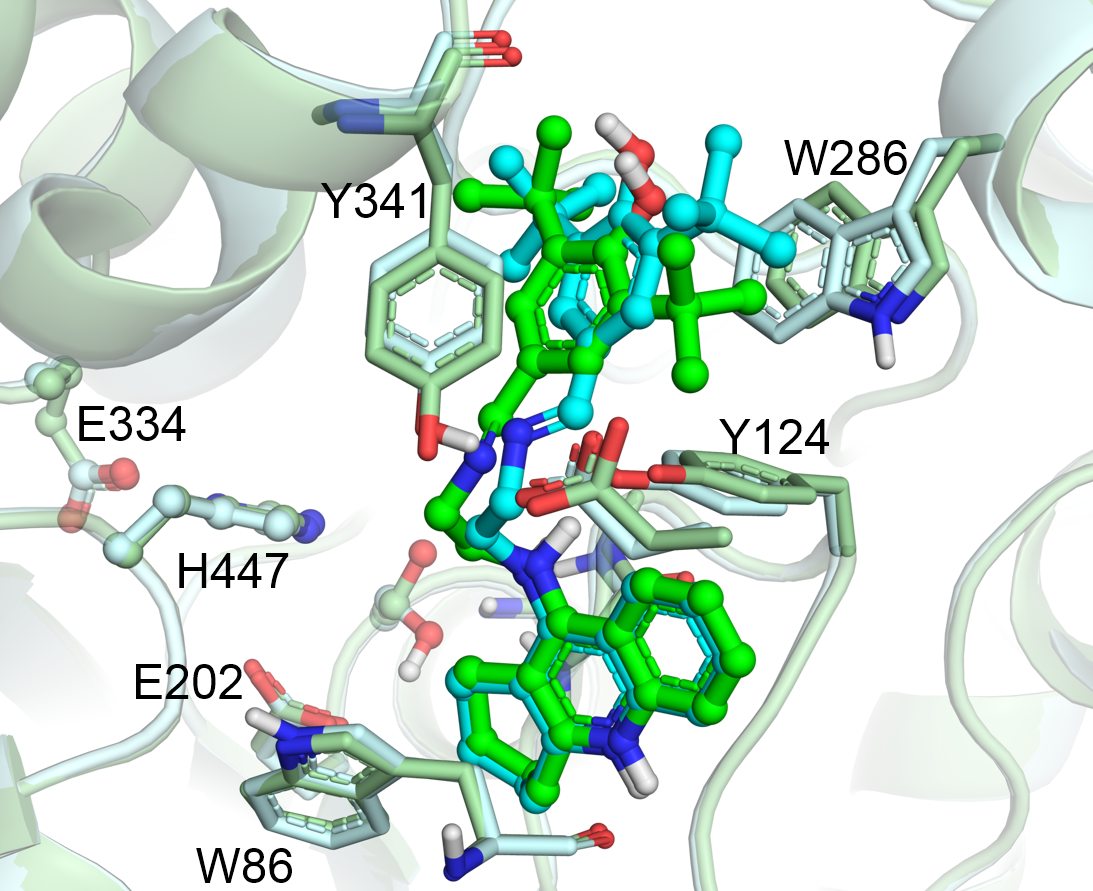 | 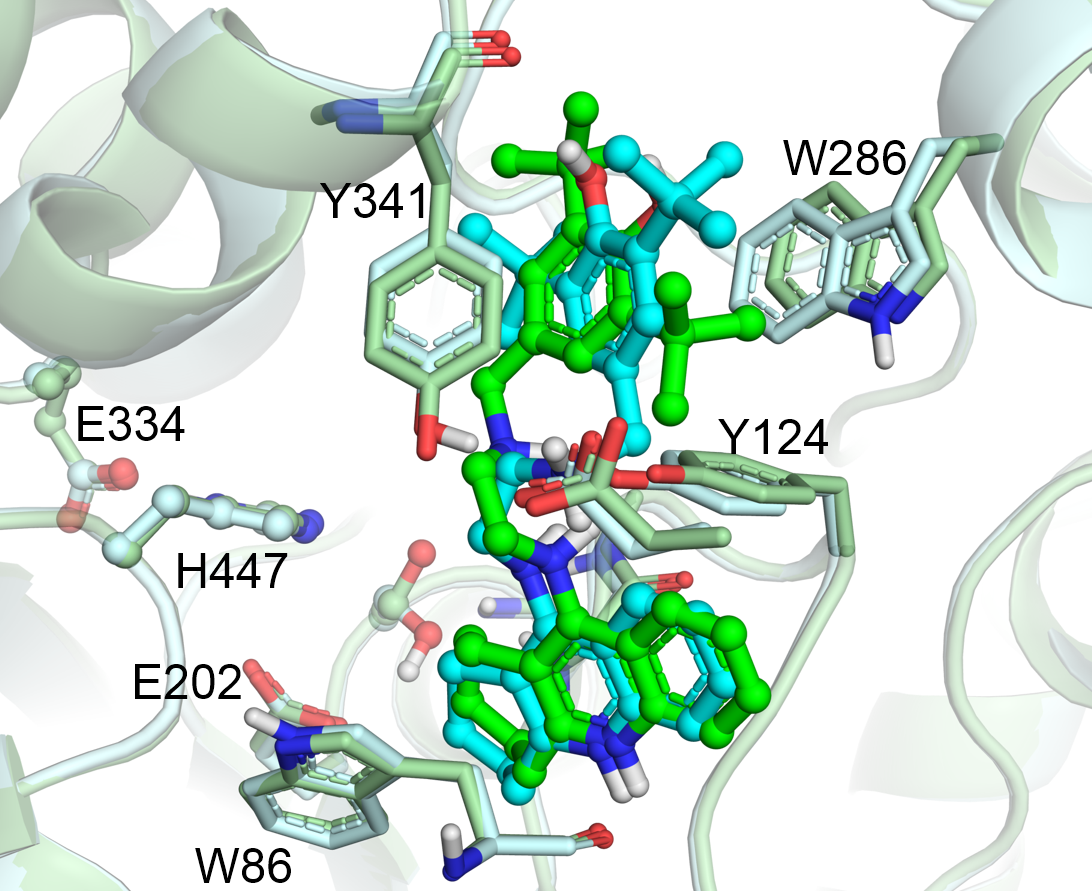 |
| --- | --- |
| **(A)** | **(B)** |

**Figure S2.** Overlay of binding poses of compounds **7c** (A) and **8c** (B) obtained by molecular docking with human AChE (PDB ID 4EY4, carbon atoms colored cyan) and mouse AChE (PDB ID 5EHQ, carbon atoms colored green).

__________

^1^The structure of the AChE inhibitor huprine W resembles somewhat the structure of tacrine. However, the available PDB X-ray structure of hAChE inhibited by huprine W (PDB ID 4BDT [5]) also contains the snake toxin fasciculin-2 (FAS-2) bound to the PAS, which limits the usability of this structure. Indeed, binding between AChE and FAS-2 induces conformational changes in the whole protein, and in particular, the entire gorge [6], which significantly affects the binding mode of dual binding site inhibitors [1]. Before the X-ray structure of the apo-state hAChE was resolved in 2012, FAS-2-bound hAChE structures were the only option, and were used along with mAChE X-ray structures for molecular docking studies. Since X-ray structures of apo-state and co-crystallized with pharmacologically important ligands hAChE were published [3], their use enables us to model interactions between hAChE and inhibitors without the effect of FAS-2 on overall protein conformation, but accounting for conformational changes induced by drugs bound in the hAChE gorge.

**References**

1. Lushchekina, S.V.; Makhaeva, G.F.; Novichkova, D.A.; Zueva, I.V.; Kovaleva, N.V.; Richardson, R.J. Supercomputer modeling of dual-site acetylcholinesterase (AChE) inhibition. *Supercomputing Frontiers and Innovations* **2018**, *5*, 89-97, doi:10.14529/jsfi1804.

2. Makhaeva, G.F.; Kovaleva, N.V.; Boltneva, N.P.; Lushchekina, S.V.; Rudakova, E.V.; Stupina, T.S.; Terentiev, A.A.; Serkov, I.V.; Proshin, A.N.; Radchenko, E.V., et al. Conjugates of tacrine and 1,2,4-thiadiazole derivatives as new potential multifunctional agents for Alzheimer's disease treatment: Synthesis, quantum-chemical characterization, molecular docking, and biological evaluation. *Bioorg. Chem.* **2020**, *94*, 103387, doi:10.1016/j.bioorg.2019.103387.

3. Cheung, J.; Rudolph, M.J.; Burshteyn, F.; Cassidy, M.S.; Gary, E.N.; Love, J.; Franklin, M.C.; Height, J.J. Structures of human acetylcholinesterase in complex with pharmacologically important ligands. *J. Med. Chem.* **2012**, *55*, 10282-10286, doi:10.1021/jm300871x.

4. Lushchekina, S.V.; Masson, P. Slow-binding inhibitors of acetylcholinesterase of medical interest. *Neuropharmacology* **2020**, *177*, 108236, doi:10.1016/j.neuropharm.2020.108236.

5. Nachon, F.; Carletti, E.; Ronco, C.; Trovaslet, M.; Nicolet, Y.; Jean, L.; Renard, P.Y. Crystal structures of human cholinesterases in complex with huprine W and tacrine: elements of specificity for anti-Alzheimer's drugs targeting acetyl- and butyryl-cholinesterase. *Biochem. J.* **2013**, *453*, 393-399, doi:10.1042/BJ20130013.

6. Bourne, Y.; Taylor, P.; Marchot, P. Acetylcholinesterase inhibition by fasciculin: crystal structure of the complex. *Cell* **1995**, *83*, 503-512, doi:10.1016/0092-8674(95)90128-0.

7. Bar-On, P.; Millard, C.B.; Harel, M.; Dvir, H.; Enz, A.; Sussman, J.L.; Silman, I. Kinetic and structural studies on the interaction of cholinesterases with the anti-Alzheimer drug rivastigmine. *Biochemistry* **2002**, *41*, 3555-3564, doi:10.1021/bi020016x.

8. Chandar, N.B.; Efremenko, I.; Silman, I.; Martin, J.M.L.; Sussman, J.L. Molecular dynamics simulations of the interaction of Mouse and Torpedo acetylcholinesterase with covalent inhibitors explain their differential reactivity: Implications for drug design. *Chem.-Biol. Interact.* **2019**, *310*, 108715, doi:10.1016/j.cbi.2019.06.028.

9. Bourne, Y.; Sharpless, K.B.; Taylor, P.; Marchot, P. Steric and Dynamic Parameters Influencing In Situ Cycloadditions to Form Triazole Inhibitors with Crystalline Acetylcholinesterase. *J. Am. Chem. Soc.* **2016**, *138*, 1611-1621, doi:10.1021/jacs.5b11384.
