# Supplementary material for: New Multifunctional Agents Based on Conjugates of 4-Amino-2,3-polymethylenequinoline and Butylated Hydroxytoluene for Alzheimer’s Disease Treatment
Source: Molecules. 2020 Dec 12;25(24):5891. doi: 10.3390/molecules25245891 (PMC7763995; doi:10.3390/molecules25245891)
Supplement: Supplementary file 1 [file molecules-25-05891-s001.zip › Supplementary/Supplemental_Title.docx]

New Multifunctional Agents Based on Conjugates of 4-Amino-2,3-polymethylenequinoline and Butylated Hydroxytoluene for Alzheimer’s Disease Treatment

Galina F. Makhaeva^1^, Nadezhda V. Kovaleva^1^, Elena V. Rudakova^1^, Natalia P. Boltneva^1^, Sofya V. Lushchekina^1,2^, Irina I. Faingold^3^, Darya A. Poletaeva^3^, Yuliya V. Soldatova^3^, Raisa A. Kotelnikova^3^, Igor V. Serkov^1^, Anatoly K. Ustinov^1^, Alexey N. Proshin^1^, Eugene V. Radchenko^1,4^, Vladimir A. Palyulin^1,4^, and Rudy J. Richardson^5,6,7,8*^

^1^ Institute of Physiologically Active Compounds Russian Academy of Sciences, Chernogolovka, 142432, Russia; gmakh@ipac.ac.ru (G.F.M.); kovalevanv@ipac.ac.ru (N.V.K.); rudakova@ipac.ac.ru (E.V.Ru.); boltneva@ipac.ac.ru (N.P.B); sofya.lushchekina@gmail.com (S.V.L); serkoviv@mail.ru (I.V.S.); ustinov_51@mail.ru (A.K.U.); proshin@ipac.ac.ru (A.N.P.); genie@qsar.chem.msu.ru (E.V.R.); vap@qsar.chem.msu.ru (V.A.P.)

^2^ Emanuel Institute of Biochemical Physics Russian Academy of Sciences, Moscow, 119334, Russia

^3^ Institute of Problems of Chemical Physics of Russian Academy of Sciences, Chernogolovka, 142432, Russia; ifaingold@mail.ru (I.I.F); dapol@icp.ac.ru (D.A.P); soldatovayv@gmail.com (Yu.V.S); rkot@icp.ac.ru (R.A.K.)

^4^ Department of Chemistry, Lomonosov Moscow State University, Moscow, 119991, Russia

^5^ Department of Environmental Health Sciences, University of Michigan, Ann Arbor, MI 48109 USA

^6^ Department of Neurology, University of Michigan, Ann Arbor, MI 48109 USA

^7^ Center of Computational Medicine and Bioinformatics, University of Michigan, Ann Arbor, MI 48109 USA

^8^ Michigan Institute for Computational Discovery and Engineering, University of Michigan, MI 48109 Ann Arbor, USA

***** Correspondence: rjrich@umich.edu; Tel.: +1-734-936-0769

**Contents**

(1) Molecular docking studies including Figures S1, S2

(2) NMR spectra for compounds **7a**–**d, 8a**, **8c**

Figure S3. NMR Spectrum for **7a**;

Figure S4. NMR Spectrum for **7b;**

Figure S5. NMR Spectrum for **7c;**

Figure S6. NMR Spectrum for **7d;**

Figure S7. NMR Spectrum for **8a;**

Figure S8. NMR Spectrum for **8c.**
