# Supplementary figures and images for: New Multifunctional Agents Based on Conjugates of 4-Amino-2,3-polymethylenequinoline and Butylated Hydroxytoluene for Alzheimer’s Disease Treatment
Source: Molecules. 2020 Dec 12;25(24):5891. doi: 10.3390/molecules25245891 (PMC7763995; doi:10.3390/molecules25245891)

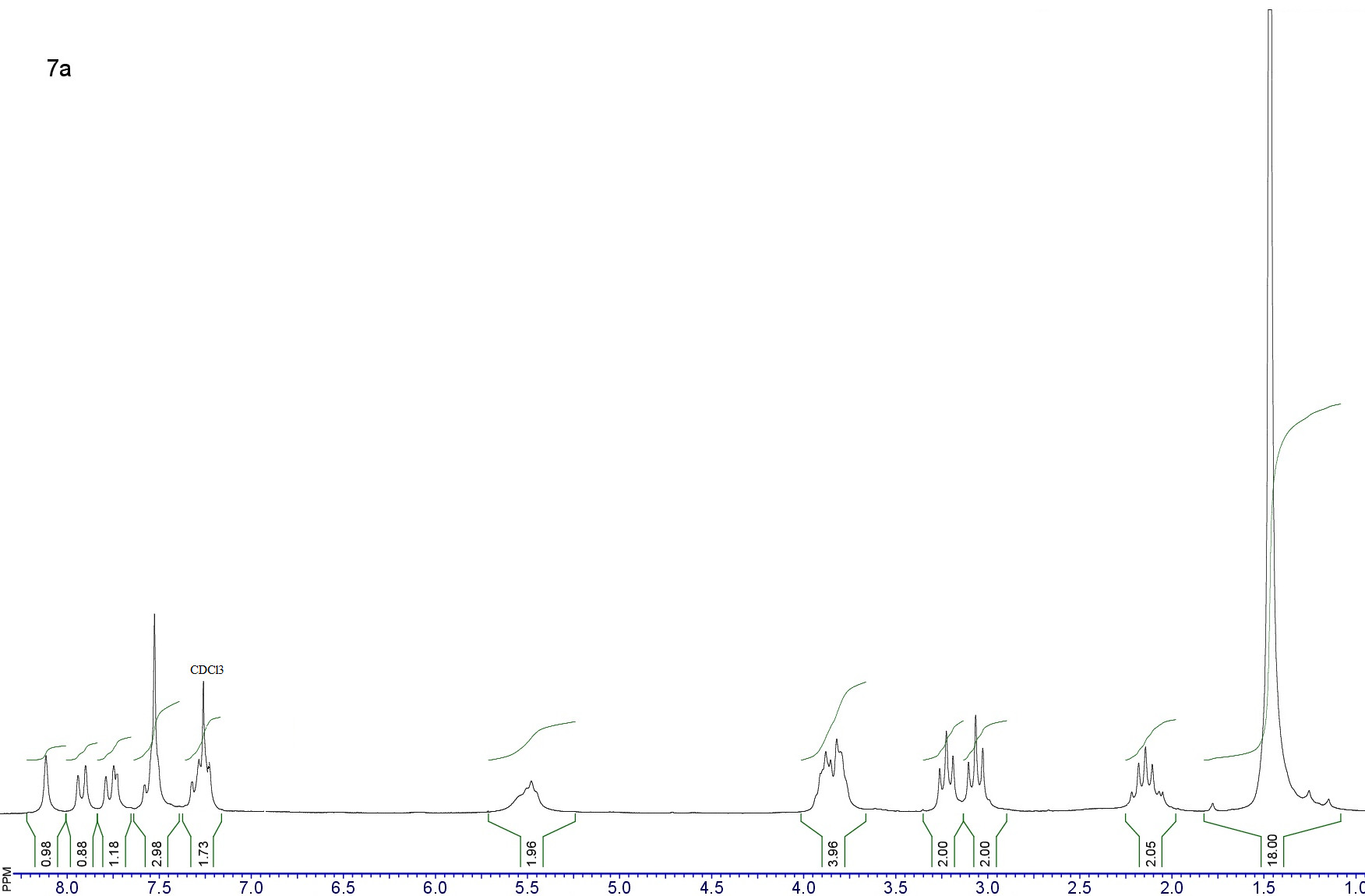


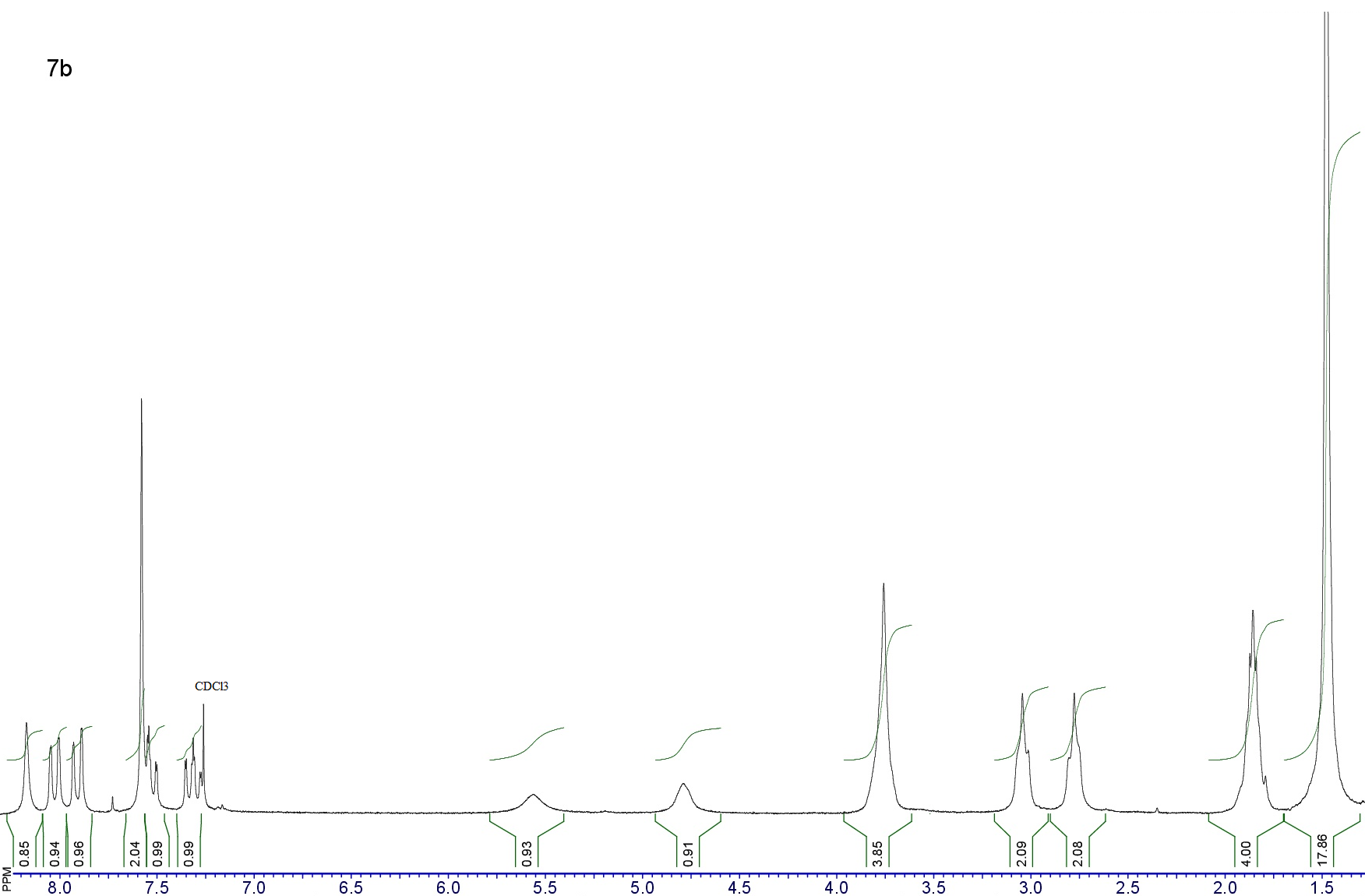


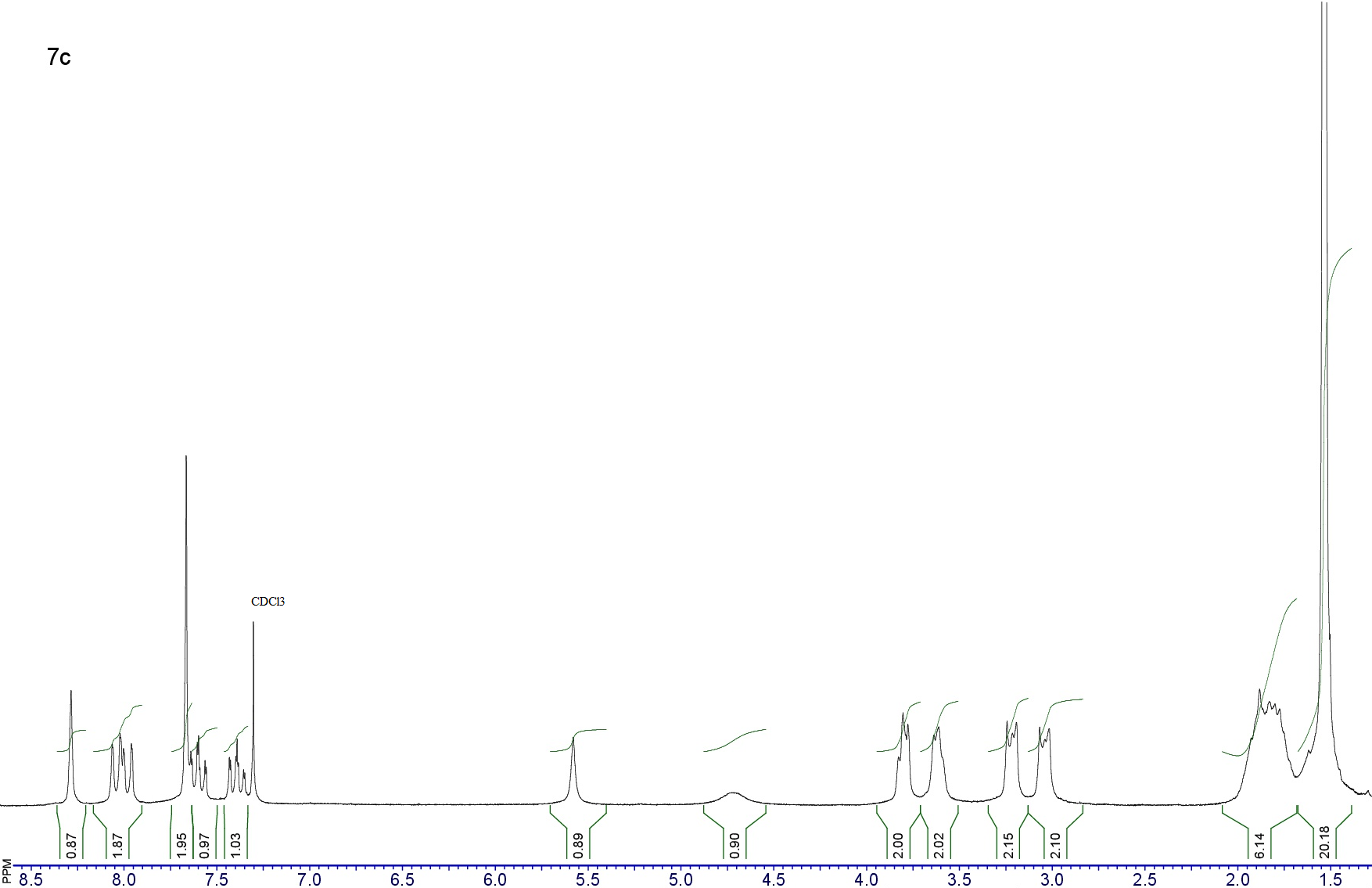


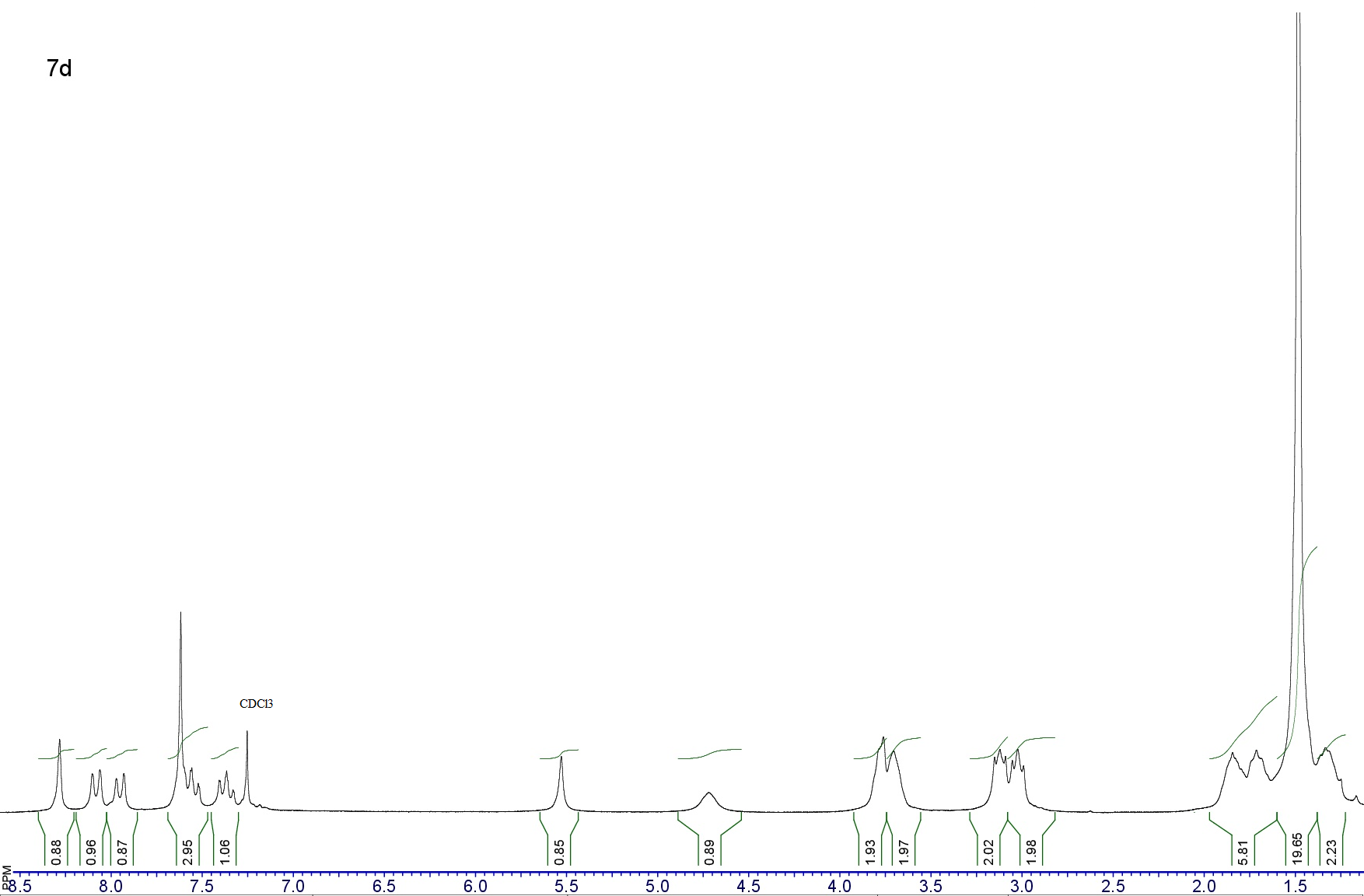


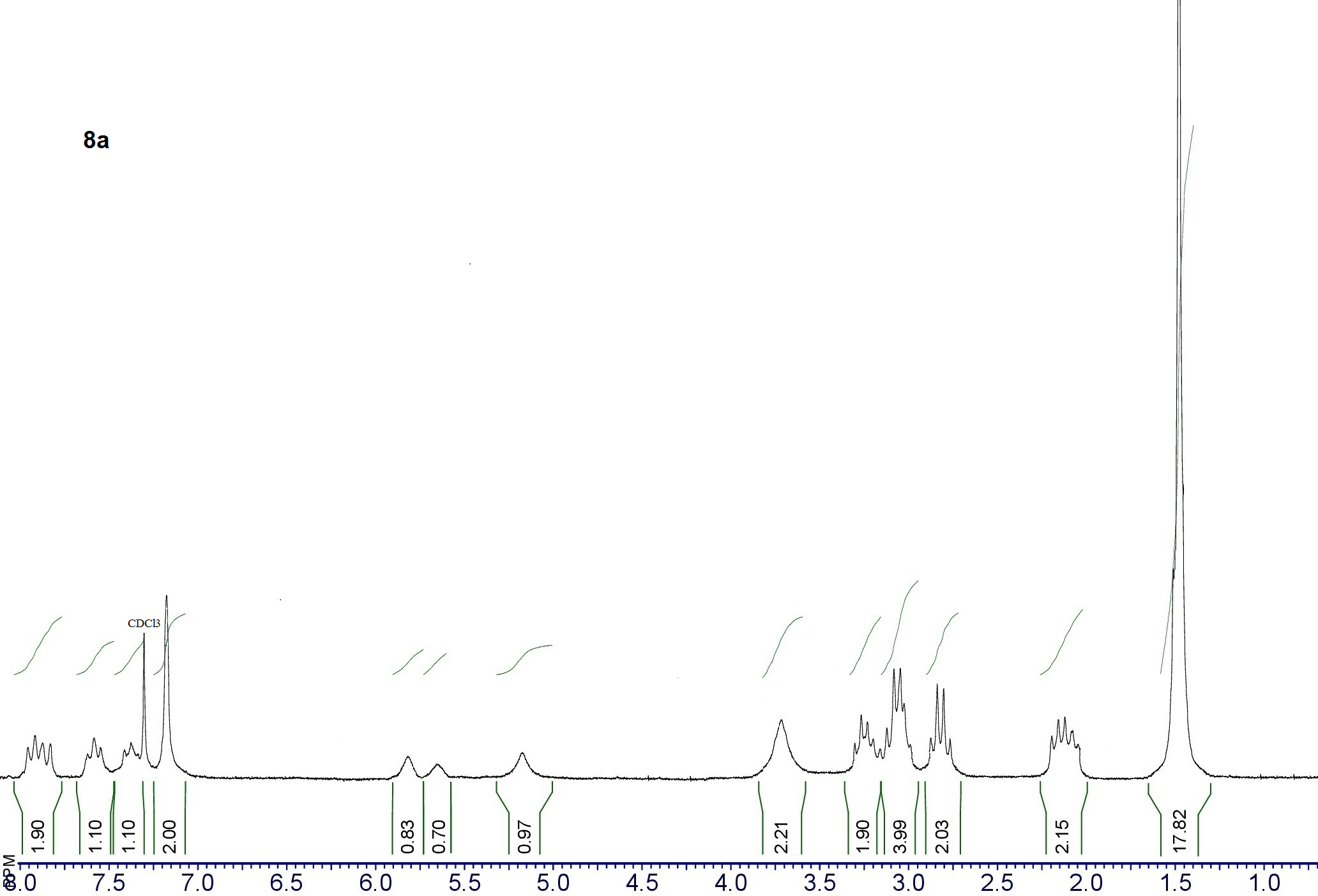


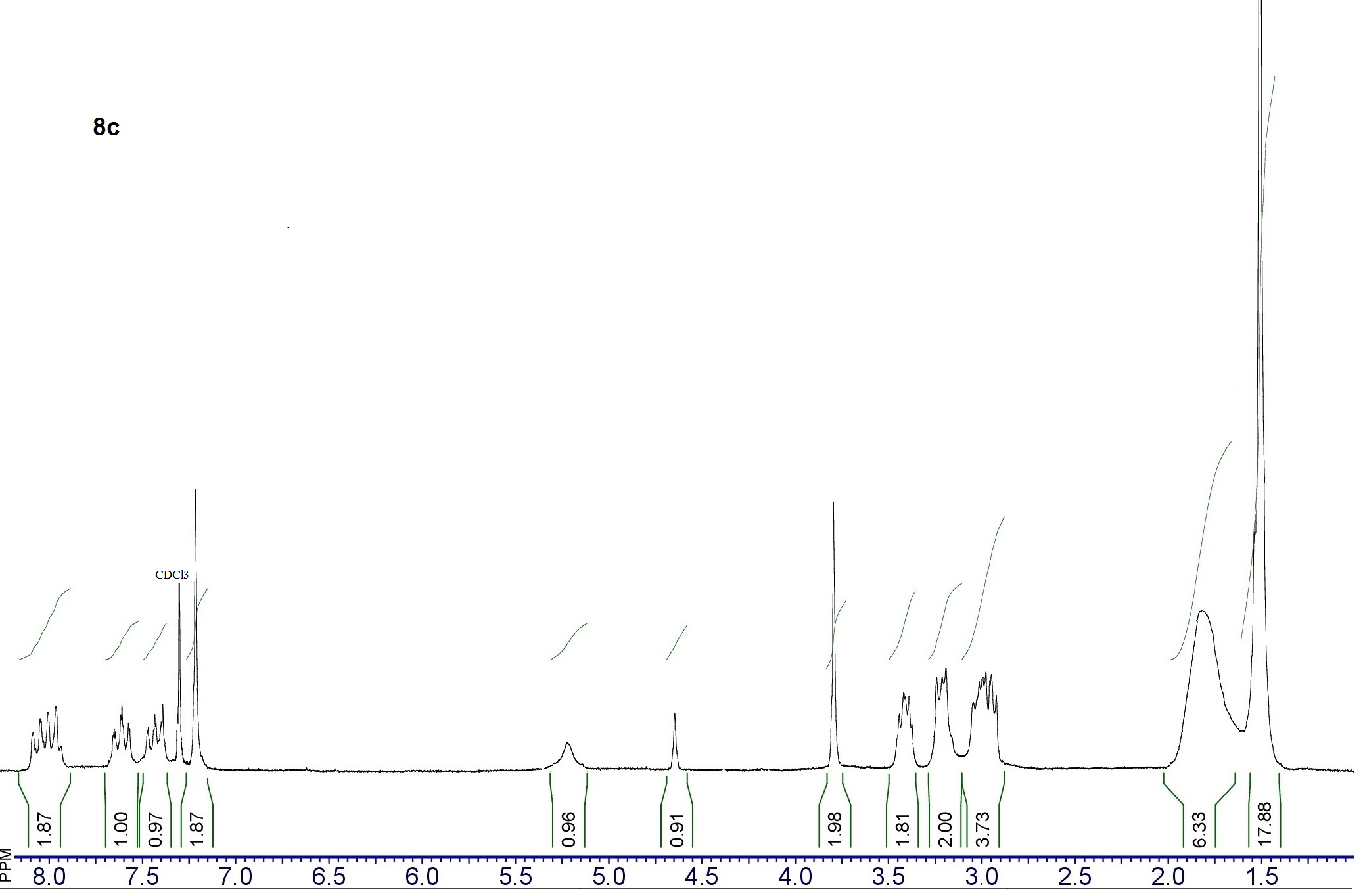

Supplement: Supplementary file 1 [file molecules-25-05891-s001.zip › Supplementary/2_NMR_7a-7d_8a&c.docx]
